# The miR-141/neuropilin-1 axis is associated with the clinicopathology and contributes to the growth and metastasis of pancreatic cancer

**DOI:** 10.1186/s12935-019-0963-2

**Published:** 2019-09-27

**Authors:** Lixin Ma, Bo Zhai, Huaqiang Zhu, Weidong Li, Wenjing Jiang, Liwang Lei, Shujun Zhang, Haiquan Qiao, Xian Jiang, Xueying Sun

**Affiliations:** 1 Department of General Surgery, The First Affiliated Hospital of Harbin Medical University, Harbin, 150001 China; 2 Department of General Surgery, The Fourth Affiliated Hospital of Harbin Medical University, Harbin, 150001 China; 3 The Hepatosplenic Surgery Center, The First Affiliated Hospital of Harbin Medical University, Harbin, 150001 China; 4 Department of Hepatobiliary Surgery, Shandong Provincial Hospital, Jinan, 250021 China; 5 Department of Pathology, The Fourth Affiliated Hospital of Harbin Medical University, Harbin, 150001 China

**Keywords:** Neuropilin-1, Pancreatic cancer, MicroRNA-141, Proliferation, Metastasis, Epithelial–mesenchymal transition, Transforming growth factor β, Cellular signaling

## Abstract

**Background:**

Neuropilin-1 (NRP-1) is a non-tyrosine kinase receptor interacting with multiple signaling pathways that underpin the biological behavior and fate of cancer cells. However, in pancreatic cancer, the mechanisms underlying the function of NRP-1 in cell proliferation and metastasis and the involvement of regulatory upstream miRNAs remain unclear.

**Methods:**

Potential miRNAs were mined by using multiple bioinformatics prediction tools and validated by luciferase assays. The expression of NRP-1 and miRNA-141 (miR-141) in pancreatic tissues and cells was examined by immunohistochemistry, immunoblotting and/or real-time RT-PCR. Stable transfected cells depleted of NRP-1 were generated, and regulatory effects of miR-141 were investigated by transfecting cells with miR-141 mimics and anti-miR-141. Assays of cell viability, proliferation, cell cycle distribution, transwell migration and cell scratch were employed. Xenograft tumor models were established to assess the effects of NRP-1 depletion on tumorigenesis and liver metastasis, and therapeutic effects of miR-141 on tumor growth. The role of miR-141/NRP-1 axis in regulating epithelial–mesenchymal transition (EMT) by co-interacting the TGF-β pathway was examined.

**Results:**

In this study, of 12 candidate miRNAs identified, miR-141 showed the strongest ability to regulate NRP-1. In pancreatic cancer tissues and cells, the expression level of NRP-1 was negatively correlated with that of miR-141. NRP-1 was highly expressed in pancreatic cancer tissues compared with normal pancreatic tissues, and its expression levels were positively correlated with tumor grade, lymph metastasis and AJCC staging. NRP-1 depletion inhibited cell proliferation by inducing cell cycle arrest at the G0/G1 phase through upregulating p27 and downregulating cyclin E and cyclin-dependent kinase 2, and reduced cell migration by inhibiting EMT through upregulating E-cadherin and downregulating Snail and N-cadherin. Through downregulating NRP-1, miR-141 mimics showed a similar effect as NRP-1 depletion on cell proliferation and migration. NRP-1 depletion suppressed tumor growth and liver metastasis and miR-141 mimics inhibited the growth of established tumors in mice. NRP-1 depletion and/or miR-141 mimics inhibited the activation of the TGF-β pathway stimulated by TGF-β ligand.

**Conclusions:**

The present results indicate that NRP-1 is negatively regulated by miR-141 and the miR-141/NRP-1 axis may serve as potentially valuable biomarkers and therapeutic targets for pancreatic cancer.

## Background

Pancreatic cancer is the fourth leading cause of cancer-related deaths and its incidence is projected to increase as the second in the United States and Europe by 2030 [1, 2]. The development of effective molecular targeting drugs is lagging far behind for pancreatic cancer, which is usually diagnosed at advanced stages with an overall 5-year survival rate as low as 5% [1]. Such a poor outcome highlights the urgent need for seeking molecular targets and exploring underlying mechanisms for combating pancreatic cancer.

Neuropilin-1 (NRP-1) is a non-tyrosine kinase transmembrane receptor and has drawn great attention in cancer research, since it is highly expressed in gastrointestinal cancers [3, 4], including pancreatic cancer [5–8]. We have recently reported that NRP-1 is overexpressed in gastric cancer [9] and cholangiocarcinoma [10], and contributes to their clinicopathology, cell growth and metastasis. More importantly, NRP-1 acts as multifunctional co-receptors interacting with vascular endothelial growth factor (VEGF), epidermal growth factor (EGF) and hepatic growth factor (HGF)-regulated cellular signaling pathways [9, 11, 12]. These pathways play key roles in the progression of pancreatic cancer [5, 6, 8, 13]. In addition, the expression level of NRP-1 correlates inversely with survival of pancreatic cancer patients [5, 7].

It estimated that microRNAs (miRNAs), small endogenous noncoding RNAs of 19 to 25 nucleotides, negatively regulate the expression of more than 60% of protein-coding genes involved in a wide range of biological processes [14, 15]. Aberrant expression of miRNAs has been widely studied in human cancers including pancreatic cancer [16]. Increasing evidence suggests that miRNAs are associated with clinicopathological characteristics and prognosis of pancreatic cancer [16, 17]. For example, miR-141 acts as a tumor suppressor by inhibiting the proliferation and invasion of pancreatic cancer cells [18, 19]. In addition, miRNA-148 and miR-124 have been shown to act as upstream suppressors of NRP-1 signaling [20, 21]. We have recently demonstrated that NRP-1 regulated by miR-320 contributes to the growth and metastasis of cholangiocarcinoma cells [10]. However, little is known about the upstream mRNAs for NRP-1 in pancreatic cancer. Therefore, the present study was designed to seek potential miRNAs that regulate NRP-1 and investigate how miRNA-regulated NRP-1 contributes to the clinicopathology, growth and metastasis of pancreatic cancer.

## Materials and methods

### Patients

The study protocol had been approved by Harbin Medical University in China and all patients had given their informed consent prior to the inclusion in the study. Formalin-fixed tumor specimens were collected from 57 patients, who underwent pancreatic resection at the First (n = 28) and Fourth (n = 29) Affiliated Hospital of Harbin Medical University from January 2016–December 2018. Among these subjects, 23 pairs of fresh pancreatic cancer tissues and adjacent normal pancreatic tissues were collected at the First (n = 15) and Fourth (n = 8) Affiliated Hospital of Harbin Medical University from January–December of 2018, and these tissues were snap frozen in liquid nitrogen. The diagnosis was pathologically confirmed as pancreatic ductal adenocarcinoma and disease was staged in accordance with the Eighth Edition of the American Joint Committee on Cancer (AJCC) TNM Staging System [22]. Other pancreatic neoplasms, such as endocrine tumor, intraductal papillary mucinous or mucinous cystic adenocarcinoma, were excluded. Patients who received preoperative radiotherapy or chemotherapy or had a history of cancer of any other type were also excluded.

### Cell lines, antibodies, reagents and kits

Human pancreatic cancer cell lines cells (BxPC-3, AsPC-1, PANC-1, MIA-PaCa-2, Capan-1 and SUIT-2) were obtained from the Type Culture Collection Cell Bank (Chinese Academy of Sciences Committee, Shanghai, China), and normal pancreatic duct epithelial HDPE6C7 cells were a gift from Prof. Huaizhi Wang (The First Hospital Affiliated to AMU, Chongqing, China). All cell lines were authenticated by short tandem repeat analysis and confirmed to be negative for mycoplasma infection by using a PCR-based Universal Mycoplasma Detection kit (American Type Culture Collection, Manassas, VA, USA). Cells were routinely cultured in Dulbecco’s Modified Eagle Medium (DMEM) (Gibco BRL, Grand Island, NY, USA) supplemented with 10% fetal bovine serum in a humidified atmosphere of 5% CO_2_. BxPC-NRP^low^ cells depleted of NRP-1 were generated from parental BxPC-3 cells as described previously [9] and in Additional file 1: Materials S1, which also lists detailed information for antibodies, reagents and kits (Additional file 1: Table S1).

### Immunohistochemistry of clinical specimens, establishment of stable transfectants depleted of NRP-1, Transfection of oligonucleotides targeting miRNAs, Cell viability analysis, EdU (5-ethynyl-2′-deoxyuridine) proliferation assay, assessment of cell cycle, transwell migration assay, cell scratch assay, quantitative reverse-transcription polymerase chain reaction (qRT-PCR), Immunoblotting analysis, Gelatin zymography assay, Immunohistochemistry of assessing gene expression in animal tumor tissues, In situ Ki-67 proliferation index and assessment of tumor vascularity

These are described in detail under Additional file 1: Materials S1 and have also been reported previously [9, 10, 23, 24].

### Animal experiments

Male nude BALB/c-nu/nu mice (aging 6–8 weeks) obtained from SLAC laboratory Animal Co., Ltd. (Shanghai, China) were maintained at the Animal Research Center of the First Affiliated Hospital of Harbin Medical University. Animal experiments were performed according to a permit (No. SYXK20020009, Harbin Medical University) in compliance with the Experimental Animal Regulations by the National Science and Technology Commission, China. Three animal models were established for investigating the role of NRP-1 on tumorigenesis and metastasis, and therapeutic effects of miR-141, respectively.

#### Subcutaneous tumor model

BxPC-3 or BxPC-NRP^low^ cells (5 × 10^6^) were injected subcutaneously into groups of 6 mice. Animals were monitored for 21 days, and then tumors were harvested, weighed and imaged.

#### Liver metastasis model

Mice were anesthetized by 1.5–3% isoflurane and underwent laparotomy. The spleen was exposed and exteriorized completely by division of the short gastric blood vessels and the gastrosplenic ligament. BxPC-3 or BxPC-NRP^low^ cells (2 × 10^5^) were slowly injected into the spleen with a 30-gauge needle. Following a delay of 5 min to allow the tumor cells to enter the portal circulation, splenectomy was performed by ligating the splenic pedicle. Mice were monitored and euthanized 4 weeks later. Livers were harvested, fixed with 4% paraformaldehyde and paraffin embedded, and transverse 10-μm sections were prepared at five different levels to cover the entire liver. Sections were stained with hematoxylin and eosin (HE), metastatic nodules containing more than six cancer cells were counted, and the number of nodules was recorded as the number of liver metastases.

#### Therapeutic effects of miR-141 in vivo

PANC-1 cells (5 × 10^6^) were injected subcutaneously into the flanks of mice. When tumors reached ~ 100 mm^3^, mice were assigned to 3 groups (n = 8 per group), which received an intratumoral injection of vehicle, negative control (NC) oligonucleotides or miR-141 mimics, respectively. The gene transfection solution was prepared by mixing oligonucleotides, Lipofectamine2000 and serum-free medium. Each tumor received an injection of 50 µl transfection solution containing 200 µg oligonucleotides. The vehicle containing the same concentrations of Lipofectamine2000 and serum-free medium served as control. Two mice from each group were sacrificed 4 days after injection for detecting gene expression. Tumors were measured and mice killed 15 days later.

### Statistical analysis

The statistical software SPSS 18.0 (SPSS 224 Inc., IL, USA) was employed for performing statistical analyses. The association between NRP-1 expression and clinicopathological parameters was analyzed by a Mann Whitey test. The correlation coefficient between the expression of NRP-1 and miR-14 was assessed by a Pearson test. Other data are expressed as mean values ± standard deviation (SD) and comparisons were made with a one-way analysis of variance (ANOVA) followed by a Dunnet’s test. *P *< 0.05 was considered statistically significant.

## Results

### Identification of potential upstream miRNAs that regulate the expression of NRP-1 in pancreatic cancer cells

**Fig. 1:**
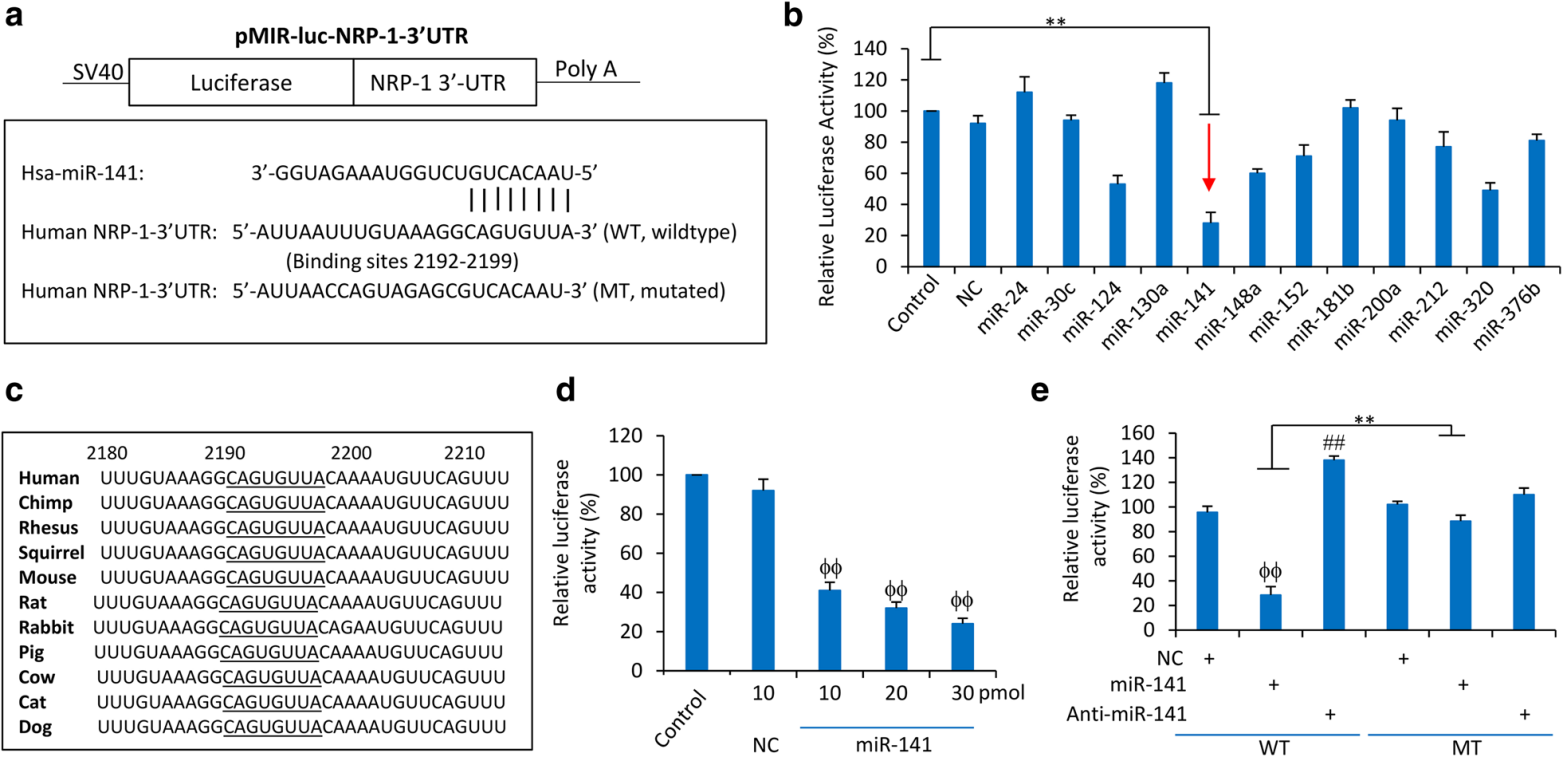
MiR-141 suppresses the expression of NRP-1 in pancreatic cancer cells by binding to the 3′UTR of NRP-1 gene. **a** Predicted binding site of Hsa-miR-141 to the 3′-UTR of the human NRP-1 gene, and the diagram of a pMIR-REPORT luciferase reporter vectors containing the wild-type (WT) or mutated (MT) 3′-UTR of NRP-1. **b** BxPC-3 cells were transfected with the WT luciferase reporter and co-transfected with negative control oligonucleotides (NC) or various miRNA mimics as indicated. Mock-transfected cells served as a control. **c** The potential binding sites of miR-141 (underlined) in the 3′UTR region of NRP-1 is highly conserved across species. **d** BxPC-3 cells were transfected with the WT luciferase reporter and co-transfected with different concentrations of miR-141 mimics. **e** BxPC-3 cells were transfected with WT or MT luciferase reporters and co-transfected with NC, miR-141 mimics or anti-miR-141. Relative luciferase activity was calculated as the percentage of luciferase activity in luciferase reporter-transfected cells over the control. “ϕϕ” (P < 0.001) indicates a significant reduction, while “##” (P < 0.001), a significant increase, compared with NC-transfected cells. “**” (P < 0.001) indicates a significant difference

We first detected the expression levels of NRP-1 protein (Additional file 1: Figure S1A, B) and mRNA (Additional file 1: Figure S1C) in a panel of human pancreatic cancer cells and pancreatic duct epithelial HDPE6C7 cells. HDPE6C7 cells expressed significantly lower levels of NRP-1 than any of the pancreatic cancer cells. We employed multiple widely used miRNA prediction programs including miRWalk (http://mirwalk.umm.uni-heidelberg.de/), TargetScan (http://www.targetscan.org/), miRanda (https://omictools.com/miranda-tool), miRTarBase (http://mirtarbase.mbc.nctu.edu.tw/) and mirdb (http://mirdb.org/) to excavate potential miRNAs that regulate NRP-1. Twelve potential miRNAs (miR-24, miR-30c, miR-124, miR-130a, miR-141, miR-148a, miR-152, miR-181b, miR-200a, miR-212, miR-320 and miR-376) were identified (Additional file 1: Figure S2). To validate the informatics prediction, we constructed a luciferase reporter vector containing full-length 3′UTR of human NRP1 mRNA (NCBI Gene ID: 8829), with expression driven by the SV40 promoter (Fig. 1a). BxPC-3 cells were transfected with the vector containing 3′UTR of NRP1 mRNA and co-transfected with each of the 12 miRNA mimics or NC oligonucleotides (Additional file 1: Table S2). Among the 12 miRNAs, miR-141 displayed the strongest ability to repress the luciferase activity (Fig. 1b). The miR-141-binding site on the 3′UTR of NRP1 is highly conserved across all the available species (Fig. 1c) and the effect of miR-141 on luciferase activity was dose-dependent (Fig. 1d). To verify the suppressive effect of miR-141 on NRP-1 expression through the predicted miR-141-binding site, a mutated version of luciferase reporter vector was generated (Fig. 1a). Reporter assays demonstrated that the miR-141-binding site was necessary for miR-141-mediated negative regulation of NRP-1 expression (Fig. 1e). The expression level of miR-141 was also negatively correlated with that of NRP-1 protein and mRNA in pancreatic cancer cells (Additional file 1: Figure S3).

### NRP-1 expression in pancreatic cancer tissues and its correlation with clinicopathological parameters

By using immunohistochemistry, we found that pancreatic cancer tissues expressed higher but variable levels of NRP-1 protein, whereas normal pancreatic tissues from adjacent areas of tumors had weak NRP-1 expression (Table 1, Additional file 1: Figure S4). The level of NRP-1 expression was significantly correlated with tumor grade (*P* = 0.015), lymph metastasis (*P* = 0.002) and AJCC staging (*P* = 0.044), and marginally (*P* = 0.087) correlated with serum levels of carbohydrate antigen (CA) 19-9, a well-accepted blood-based biomarker for pancreatic cancer [25], but not with patients’ gender and age, tumor location or size (Table 1).

**Table 1:** Correlation between NRP-1 expression and clinicopathological parameters in patients with pancreatic cancer

Parameters	Total (n = 57)	NRP-1 expression level		*P* value
		Low (n = 34)	High (n = 23)	
Gender				0.862
Male	31	19	12	
Female	26	15	11	
Age (year)				0.511
< 60	35	21	14	
≥ 60	22	13	9	
Tumor grade				0.015
Well	7	6	1	
Moderate	47	26	21	
Poor	3	1	2	
Tumor location				0.396
Head	43	28	15	
Body/tail	14	6	8	
Tumor size				0.224
< 40 mm	36	22	14	
≥ 40 mm	21	12	9	
T stage				0.109
T1–T2	7	3	4	
T3	28	17	11	
T4	23	14	9	
Lymph metastasis				0.002
Negative	16	14	2	
Positive	41	20	21	
AJCC staging				0.044
I + II	26	20	6	
III + IV	31	14	17	
CA19-9 (U/L)				0.087
< 37	22	18	4	
≥ 37	35	16	19	

P value was estimated by a Mann–Whitney test

*AJCC* American Joint Committee on Cancer, *NRP-1* neuropilin-1, *CA19-9* carbohydrate antigen19-9

### NRP-1 expression negatively correlates with miR-141 in pancreatic cancer tissues

**Fig. 2:**
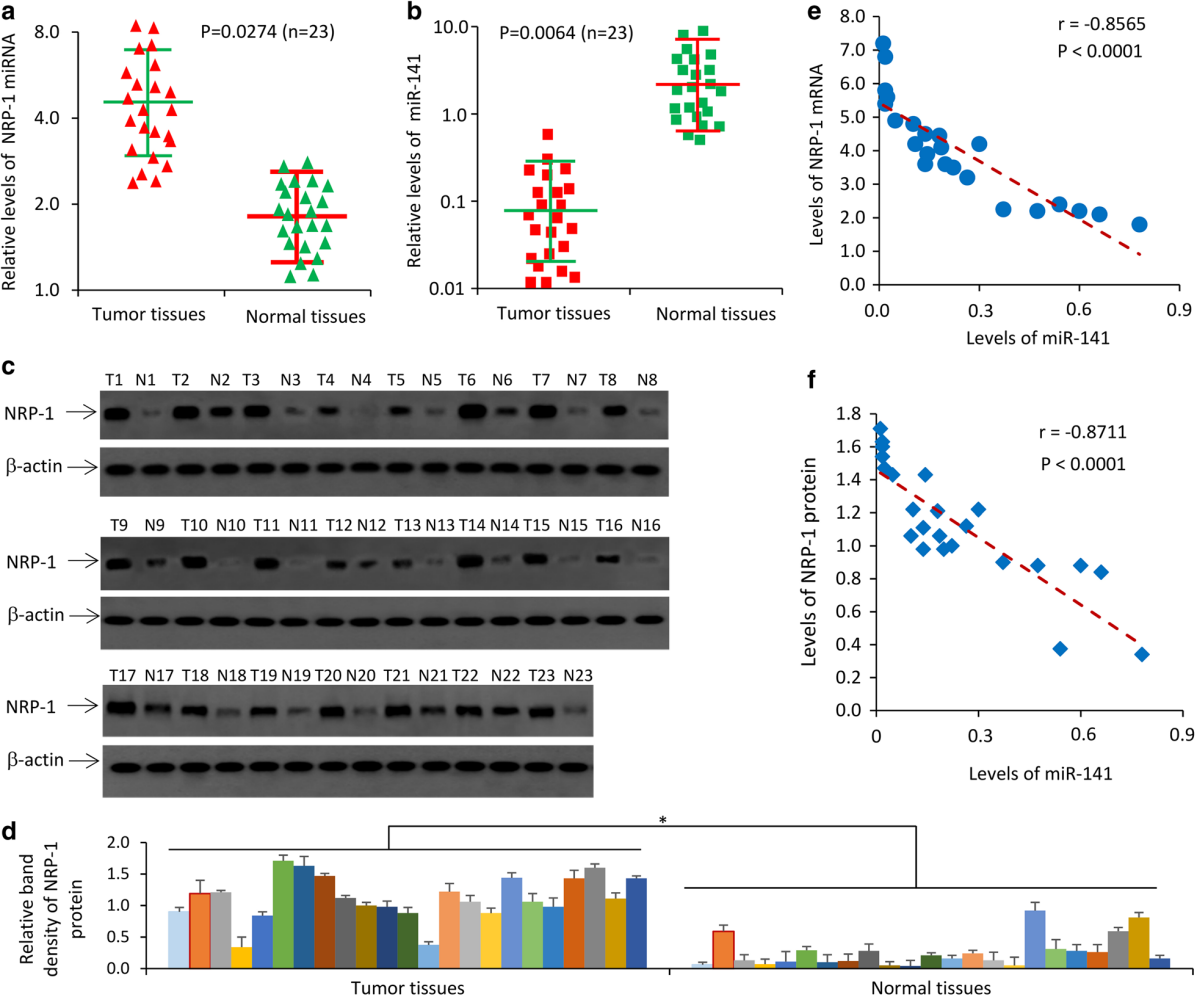
The expression of NRP-1 negatively correlates with miR-141 in pancreatic cancer tissues. **a**–**d** Twenty-three pairs of pancreatic tumor (T) and corresponding adjacent normal pancreatic (N) tissues were subjected to qRT-PCR to measure the levels of NRP-1 mRNA (**a**) and miR-141 (**b**), and were subjected to immunoblotting to detect the expression of NRP-1 protein (**c**). The density of each band was normalized to β-actin (**d**) and represented as the expression level of NRP-1 protein. “*” (P < 0.05) indicates a significant difference. **e**, **f** The correlation of the expression of NRP-1 mRNA (**e**) and protein (**f**) and miR-141 was analyzed by using a Pearson test. The correlation coefficient is denoted by “r”

We further performed qRT-PCR analyses to detect the expression levels of NRP-1 miRNA and miR-141 in 23 pairs of frozen pancreatic cancer tissues and corresponding adjacent normal pancreatic tissues. Pancreatic cancer tissues expressed significantly higher levels of NRP-1 mRNA (Fig. 2a) and significantly lower levels of miR-141 (Fig. 2b), compared with adjacent normal pancreatic tissues. The expression levels of NRP-1 in clinical tissues were confirmed by quantification of NRP-1 protein bands from immunoblotting analysis (Fig. 2c, d). The expression of both NRP-1 mRNA (Fig. 2e) and protein (Fig. 2f) was negatively correlated with that of miR-141 in pancreatic cancer tissues by using a Pearson test.

### NRP-1 regulated by miR-141 promotes the proliferation of pancreatic cancer cells

We genetically modified BxPC-3 cells, which were shown to express the highest level of NRP-1 among available pancreatic cancer cell lines (Additional file 1: Figure S1), by transfecting them with an NRP-1 shRNA or a scrambled control (Sc) shRNA vector, generating BxPC-NRP^low^ or BxPC-Sc cells, respectively. BxPC-Sc cells expressed a similar level of NRP-1, but BxPC-NRP^low^ cells expressed a significantly lower level of NRP-1, compared with parental BxPC-3 cells as detected by immunocytochemistry (Additional file 1: Figure S5A) and immunoblotting (Additional file 1: Figure S5B). Depletion of NRP-1 had little effect on the expression of miR-141, since all the above three types of cells expressed similar levels of miR-141 (Additional file 1: Figure S5C). Transfection of miR-141 mimics downregulated the expression of NRP-1 in BxPC-3 cells, and anti-miR-141 showed a slight increasing effect on the expression of NRP-1 in BxPC-NRP^low^ cells (Additional file 1: Figure S5D).

**Fig. 3:**
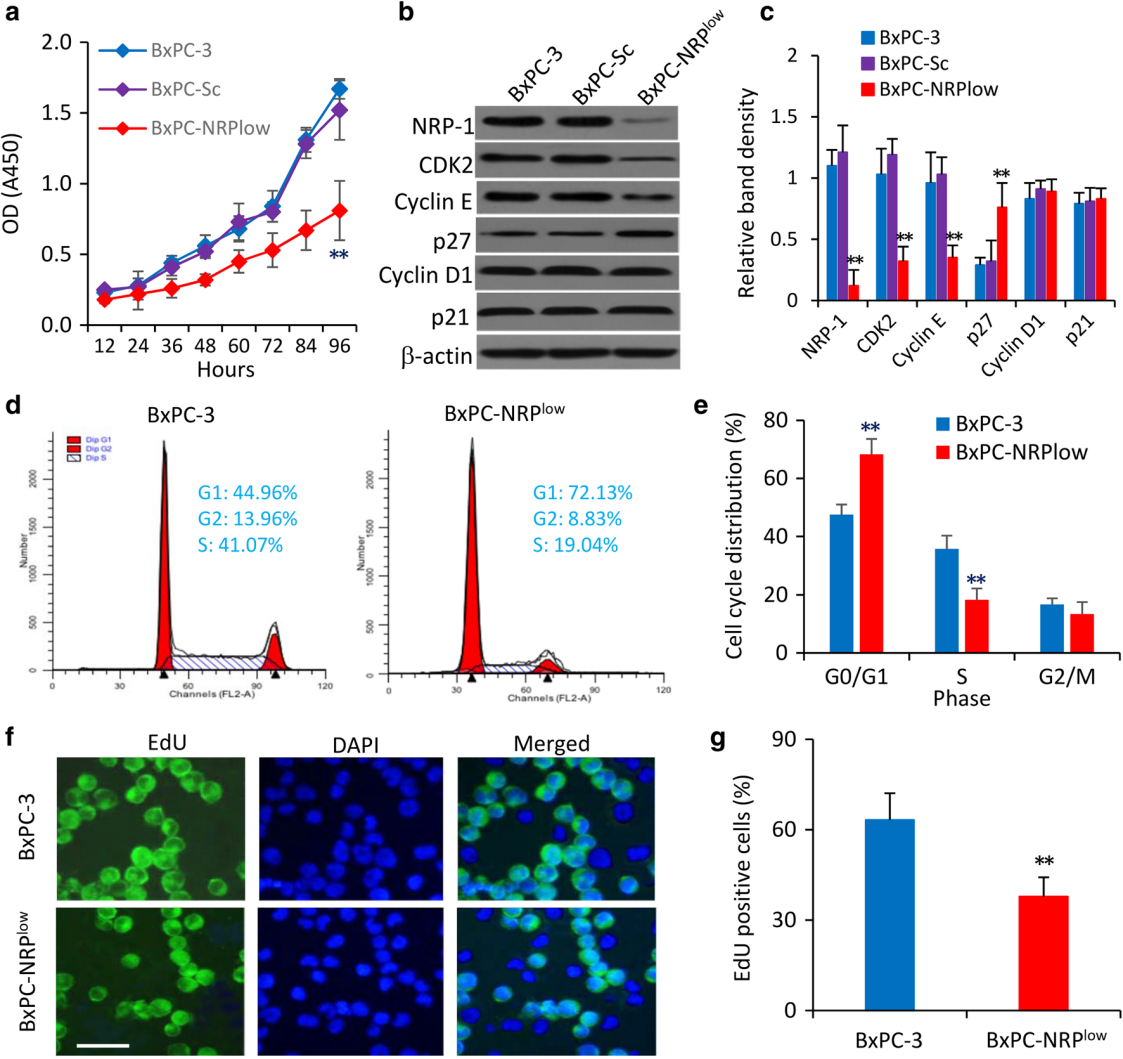
NRP-1 promotes the proliferation of pancreatic cancer cells in vitro. **a** BxPC-3, BxPC-Sc and BxPC-NRP^low^ cells were cultured for 96 h and cell viability was measured at indicated time points. **b**, **c** The above cells harvested after a 48-h culture were subjected to immunoblotting for detecting protein expression as indicated (**b**), and the density of each band was normalized to β-actin (**c**). **d**, **e** BxPC-3 and BxPC-NRP^low^ cells harvested 48 h after culturing were subjected to flow cytometry for measuring cell cycle distribution (**d**) and percentages of cells at different phases were plotted (**e**). **f**, **g** The above cells were stained by EdU (5-ethynyl-2′-deoxyuridine). **f** Representative images were from EdU-stained nuclei of proliferating cells (green) and DAPI-stained nuclei of all cells (blue), and merged photographs. Magnification bar = 20 µm. **g** The percentage of EdU-positive cells was plotted. “**” (P < 0.001) indicates a significant difference from BxPC-3 cells

BxPC-NRP^low^ cells had a significantly lower viability, while BxPC-Sc cells had a similar viability, compared with parental BxPC-3 cells (Fig. 3a). We next examined the expression of cyclin-dependent kinase 2 (CDK2), cyclin E, p27, cyclin D1 and p21, which are key proteins involved in cell proliferation and cycle progression [26]. Compared with parental BxPC-3 cells, BxPC-NRP^low^ cells expressed significantly lower levels of NRP-1, CDK2 and cyclin E, and a higher level of p27; while BxPC-Sc cells expressed similar levels of the above proteins compared with parental cells; and depletion of NRP-1 had little effects on the expression of cyclin D1 and p21 (Fig. 3b, c). Cell cycle distribution assays by flow cytometry showed that 68.3% of BxPC-NRP^low^ cells were at G0/G1 phase, while BxPC-3 cells had a lower rate (47.5%) (Fig. 3d, e). By using an EdU proliferation assay, we found that 37.8% of BxPC-NRP^low^ cells were EdU-positive, and was significantly lower than that of BxPC-3 cells (63.2%) (Fig. 3f, g).

In addition, transfection of miR-141 inhibited the proliferation of BxPC-3 cells, and anti-miR-141 partially abolished the reduced proliferation of BxPC-NRP^low^ cells by NRP-1 depletion (Additional file 1: Figure S6A). The effects of miR-141 on cell proliferation were supported by the alterations of expression of CDK2, cyclin E and p27 (Additional file 1: Figure S6B).

### NRP-1 promotes cell migration by regulating epithelial-mesenchymal transition

**Fig. 4:**
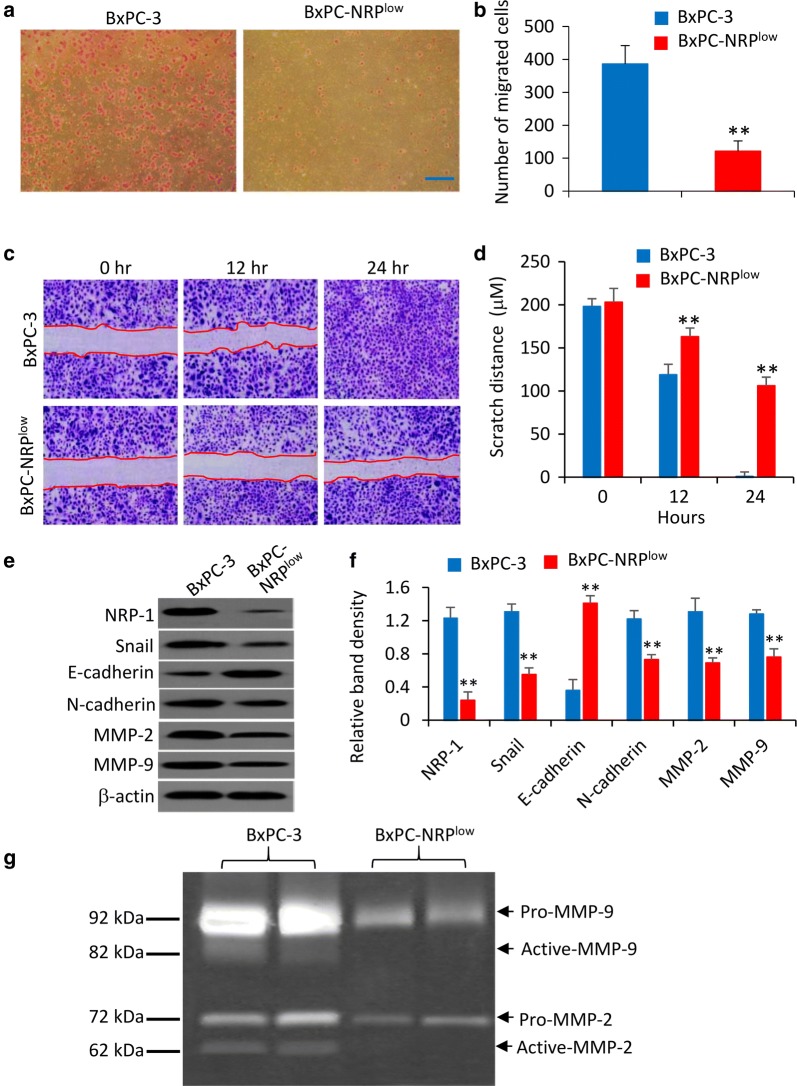
NRP-1 promotes the migration of pancreatic cancer cells by regulating EMT. **a**, **b** BxPC-3 and BxPC-NRP^low^ cells were subjected to Transwell migration assays. **a** Migrated cells were visualized using Giemsa staining. Magnification bar = 200 µm. **b** Numbers of migrating cells were counted. **c**, **d** Cells were subjected to scratch migration assays. Scratch areas were recorded (**c**) and scratch distances were quantified (**d**) at indicated time points. **e**, **f** Cells were immunoblotted for detecting key EMT proteins (**e**) and the density of each band was normalized to β-actin (**f**). **g** Cells were subjected to gelatin zymography assays for analyzing the gelatinolytic activity of MMP-9 and MMP-2. “**” (P < 0.001) indicates a significant difference from BxPC-3 cells

The number of migrated BxPC-NRP^low^ cells was significantly lower than that of parental BxPC-3 cells (Fig. 4a, b), which was supported by scratch cell assays (Fig. 4c, d). The effect of NRP-1 depletion on cell migration was confirmed in PANC-1 cells (Additional file 1: Figure S7), which were also shown to express high levels of NRP-1 (Additional file 1: Figure S1) and originally derived from human metastatic pancreatic tumor [27]. Epithelial-to-mesenchymal transition (EMT) is a critical even for the migration and metastasis of pancreatic cancer cells [28]. Therefore, we examined the effects of NRP-1 depletion on the expression of Snail, E-cadherin, N-cadherin, matrix metalloproteinase (MMP)-2 and -9 because Snail is an important EMT inducer and E-cadherin downregulation and N-cadherin upregulation are two well-known hallmarks of EMT, which correlates with the increased expression of MMP-2 and MMP-9 [29]. Here we showed that BxPC-NRP^low^ cells expressed significantly lower levels of Snail, N-cadherin, MMP-2 and MMP-9, and higher levels of E-cadherin than parental BxPC-3 cells (Fig. 4e, f). The activities of MMP-2 and MMP-9 were also reduced upon NRP-1 depletion as detected by gelatin zymography assays (Fig. 4g). The results indicate that NRP-1 may promote the migration of pancreatic cancer cells by enhancing EMT.

### NRP-1 depletion inhibits tumor growth and liver metastasis

**Fig. 5:**
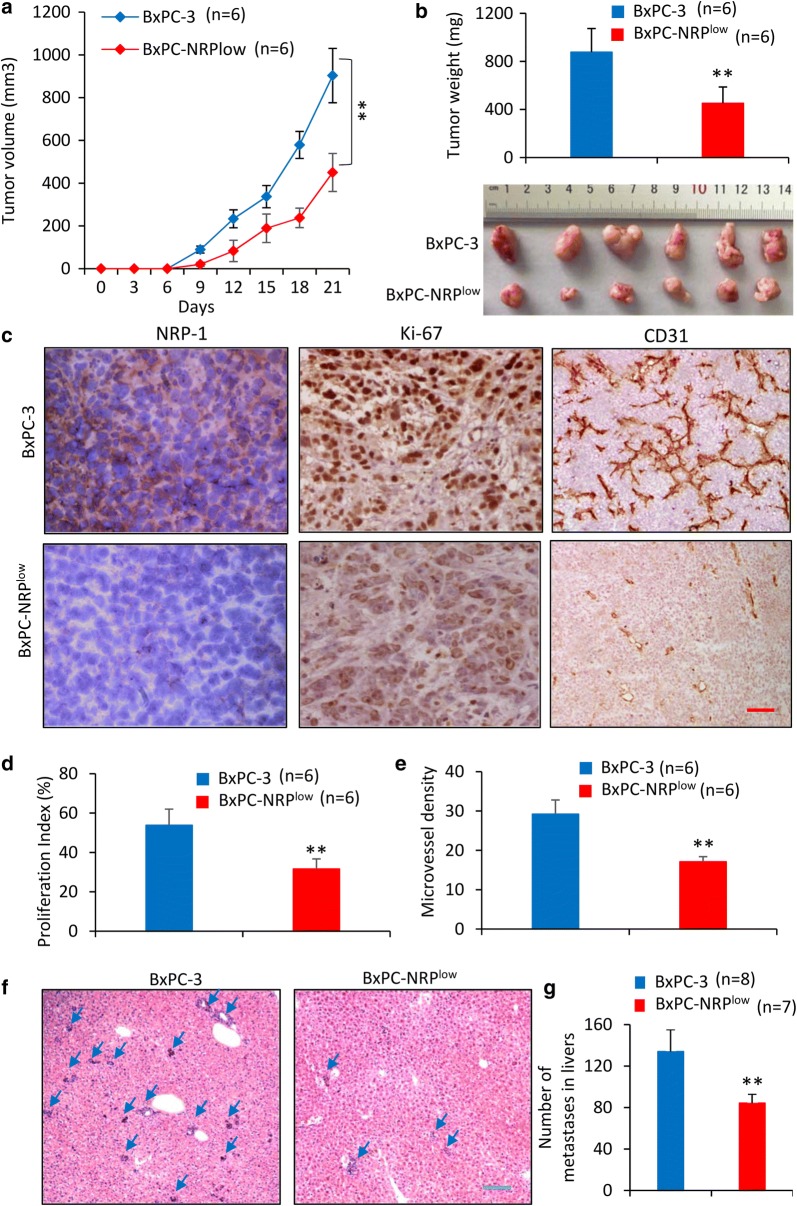
NRP-1 contributes to the growth and liver metastasis of pancreatic cancer cells in vivo. **a**, **b** BxPC-3 and BxPC-NRP^low^ cells were subcutaneously injected into the mice. **a** The growth curve of tumors were recorded. **b** Tumors were harvested, weighed and photographed at the end of experiments. **c** Illustrated are representative tumor sections immunostained by Abs against NRP-1, Ki-67 and CD31, respectively. Magnification ×400. Magnification bar = 200 µm. The cell proliferation index (**d**) and microvessel density (**e**) were quantified. **f**, **g** BxPC-3 and BxPC-NRP^low^ cells were injected into the spleen of mice, which were killed 4 weeks later to harvest the livers. **f** Illustrated are representative images of HE-stained liver sections. Magnification bar = 500 µm. Arrows point to metastatic nodules in the livers. **g** Numbers of metastatic tumor nodules in livers were counted. “n”, number of mice. “**” (P < 0.001) indicates a significant difference from mice injected with BxPC-3 cells

Subcutaneous BxPC-3 and BxPC-NRP^low^ tumors were established in mice. BxPC-3 tumors showed a significant faster growth rate than BxPC-NRP^low^ tumors (Fig. 5a). At the end of experiments, BxPC-3 tumors grew to 903.2 ± 126.5 mm^3^ (876.4 ± 195.3 mg in weight), whereas BxPC-NRP^low^ tumors were only of 450.1 ± 88.6 mm^3^ in size (451.8 ± 130.5 mg in weight) (Fig. 5b, c). In agreement with the in vitro results (Fig. 3b), BxPC-NRP^low^ tumors expressed lower levels of NRP-1 protein than BxPC-3 tumors as detected by immunohistochemistry (Fig. 5c). NRP-1 depletion significantly inhibited cell proliferation in situ (Fig. 5c, d) and reduced tumor vasculature (Fig. 5c, e).

We next established liver metastasis of pancreatic cancer cells by intra-splenic injection. Four weeks after the commencement of experiments, mouse livers were harvested, sectioned, HE-stained and metastatic nodules were counted. As shown in Fig. 5f, g, mice intra-splenically injected with BxPC-NRP^low^ cells had significantly fewer metastatic nodules in their livers (with an average number of 84.2 ± 9.1), compared with those injected with BxPC-3 cells (with an average number of 134.6 ± 20.3).

### MiR-141 participates in cell migration by regulating NRP-1 that co-interacts with TGF-β to activate the TGF-β signaling pathway

**Fig. 6:**
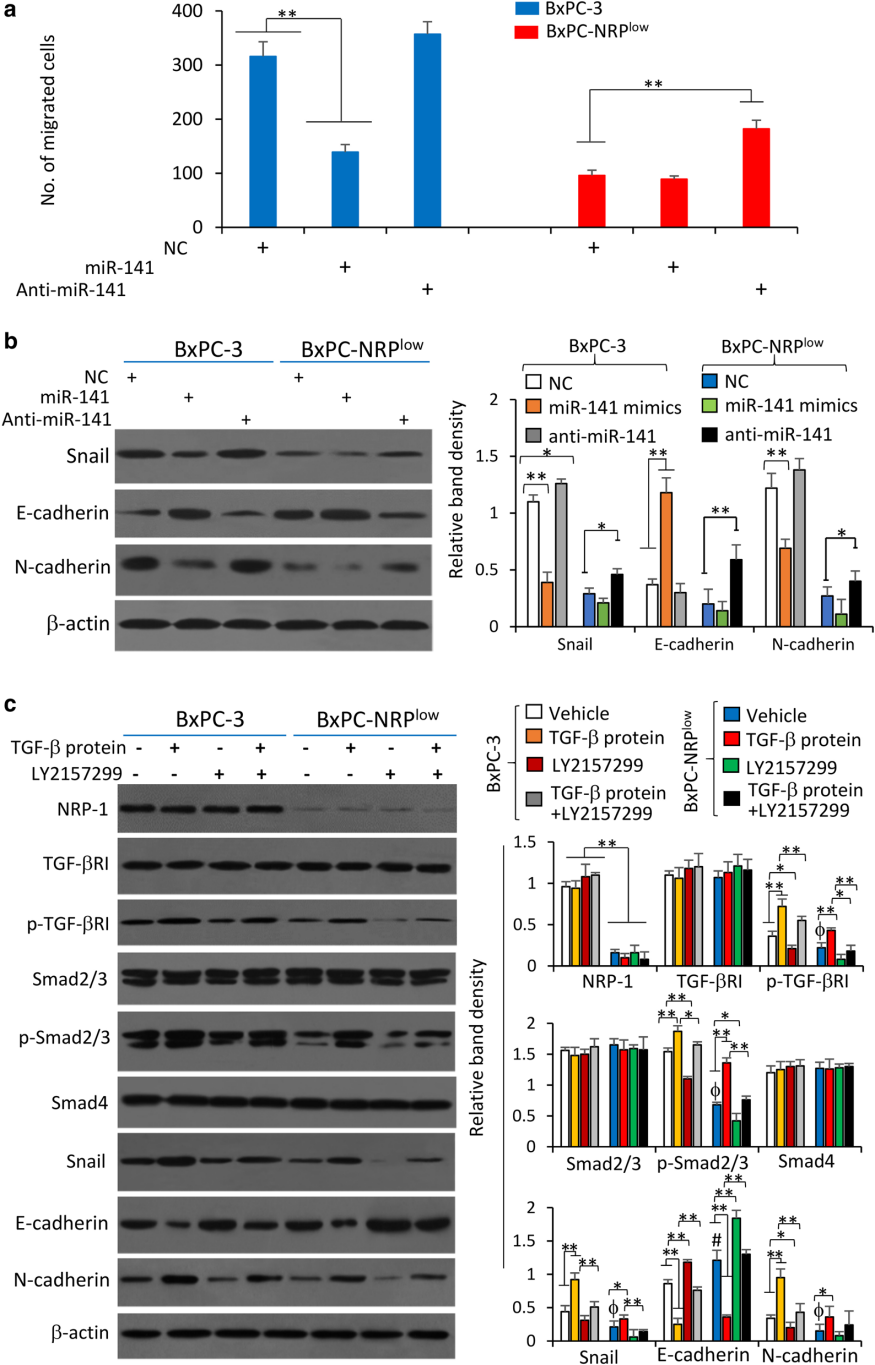
MiR-141 inhibits cell migration and EMT by dysregulating NRP-1 that co-interacts with TGF-β ligand to activate the TGF-β signaling pathway. **a**, **b** BxPC-3 and BxPC-NRP^low^ cells were transfected with negative control (NC), miR-141 mimics or anti-miR-141 oligonucleotides for 48 h. Cells were then subjected to Transwell migration assays and numbers of migrating cells were recorded (**a**), or immunoblotting for detecting key EMT proteins (**b**). **c** BxPC-3 and BxPC-NRP^low^ cells were incubated in the presence or absence of recombinant TGF-β protein (5 ng/ml) or LY2157299 (10 μg/ml) for 24 h, and subjected to immunoblotting. The density of each band was normalized to β-actin. “*” (P < 0.05) and “**” (P < 0.001) indicate a significant difference. “ϕ” (P < 0.05) indicates a significant reduction, while “#” (P < 0.05), a significant increase, from Vehicle-treated BxPC-3 cells

BxPC-3 and BxPC-NRP^low^ cells transfected with miR-141 mimics or anti-miR-141 were subjected to transwell migration assays. As shown in Fig. 6a, miR-141 transfection significantly reduced the number of migrated BxPC-3 cells, while anti-miR141 could partially restore the migrating ability of BxPC-NRP^low^ cells, which had lower migrating ability than parental BxPC-3 cells (Fig. 4). Transfection of miR-141 mimics was shown to reduce the expression of Snail and N-cadherin and increase the expression of E-cadherin in BxPC-3 cells; while anti-miR-141 transfection led to upregulation of Snail and N-cadherin and downregulation of E-cadherin in BxPC-NRP^low^ cells (Fig. 6b).

NRP-1 has been demonstrated to be a co-receptor interacting TGF-β [30] and the activation of TGF-β pathway promotes EMT of pancreatic cancer cells [31]. Therefore, we investigated whether depletion of NRP-1 could suppress the activation of TGF-β pathway stimulated by TGF-β ligand in pancreatic cancer cells. BxPC-3 and BxPC-NRP^low^ cells were incubated with recombinant human TGF-β protein or/and LY2157299, a specific TGF-β receptor (TGF-βR) inhibitor [32]. Incubation of TGF-β protein or LY2157299 had no effect on the expression of NRP-1 or TGF-βRI (Fig. 6c). However, stimulation by TGF-β led to the upregulation of p-TGF-βRI, while LY2157299 reduced the expression of p-TGF-βRI, in either BxPC-3 or BxPC-NRP^low^ cells. Depletion of NRP-1 had little effect on TGF-βRI expression, but significantly downregulated the expression of p-TGF-βRI (Fig. 6c). The alteration of TGF-β signaling activation by TGF-β protein or LY2157299 or NRP-1 depletion had little effects on the expression of Smad2/3 or Samd4, but resulted in significant changes of p-Smad2/3 expression and sequential alteration of Snail, E-cadherin and N-cadherin expression (Fig. 6c). Specifically, the activation of TGF-β signaling pathway by TGF-β protein increased the expression of p-Smad2/3, Snail and N-cadherin, while LY2157299 and NRP-1 depletion showed opposite effects and could abolish the activating effects of TGF-β protein (Fig. 6c).

### MiR-141 inhibits the growth of pancreatic tumors in mice by downregulating NRP-1

Given the negative regulatory role of miR-141 on NRP-1 expression and the inhibitory effects of NRP-1 depletion on pancreatic cancer as shown above, we further investigated whether miR-141 could act as a tumor suppressor on pancreatic tumors. Subcutaneous tumors were established in mice by inoculation of PANC-1 cells, which were also shown to express high levels of NRP-1 (Additional file 1: Figure S1). When tumors reached ~ 100 mm^3^, they were injected with either vehicle, or NC oligonucleotides, or miR-141 mimics, respectively. Tumors treated with miR-141 mimics were significantly smaller (741.7 ± 88.0 mm^3^) than vehicle-injected tumors (1324.5 ± 109.8 mm^3^), or tumors injected with NC oligonucleotides (1215.7 ± 56.6 mm^3^), 15 days after treatment commencement (Additional file 1: Figure S8A). Immunohistochemical analysis of tumors harvested 4 days after gene injection confirmed the downregulation of NRP-1 in miR-141 mimics-injected tumors (Additional file 1: Figure S8B). In consistent with the in vitro results (Additional file 1: Figure S6 and Fig. 6b), immunoblotting analysis of tumor homogenates showed that miR-141 mimics induced upregulation of p27 and E-cadherin, and downregulation of NRP-1, CDK2, cyclin E, Snail and N-cadherin (Additional file 1: Figure S8C).

## Discussion

The role of NRP-1 in cancer progression has been shown in many types of cancer including pancreatic cancer [3–10]. In this study, we have further confirmed the high expression level of NRP-1 in clinical pancreatic cancer tissues and its correlation with several clinicopathological characteristics. In investigating the regulatory upstream miRNAs, we have for the first time revealed that the high expression of NRP-1 in pancreatic cancer is attributed to the lower expression of miR-141, which negatively regulates the expression of NRP-1 by binding to the 3′-UTR of NRP-1 gene. In agreement, a low expression of miR-141 has been reported in pancreatic cancer tissues and its associations with a poor prognosis and clinicopathology have been investigated [19, 33]. MiR-141 exerts a tumor suppressing function in some other cancer types including renal cancer [34], prostate cancer [35] and breast cancer [36], although it displays its action by targeting different genes. To our knowledge, this is the first convincing study demonstrating a regulatory link between miR-141 and NRP-1 and unravelling some of the resulting downstream mechanisms in pancreatic cancer.

We have previously demonstrated that NRP-1 acts as a multiple co-receptor to promote the proliferation of cancer cells by activating the VEGF/VEGFR2 (VEGF receptor 2), EGF/EGFR (EGF receptor) and HGF/c-Met pathways [9, 10]. The VEGF/NRP-1 pathway is involved in the proliferation of cancer cells by activating Akt, leading to sequential downregulation of p27 [37], which binds to CDK2 and suppresses the activity of CDK2/cyclin E complex [38]. The cyclin E/CDK2 complex triggers the initiation of DNA replication and promotes cell cycle progression from G_1_ to S phase [38]. EGFR is highly conserved in pancreatic cancer tissues and EGFR inhibition has been shown to be an effective therapeutic strategy against pancreatic cancer [39], since the EGF/EGFR pathway activates downstream pro-oncogenic signaling pathways, resulting in proliferation and metastasis of cancer cells [40]. Upon co-stimulation of NRP-1, the activated HGF/c-Met pathway can also induce p27 downregulation [38]. We have further shown that miR-141 could inhibit the proliferation of pancreatic cancer cells by upregulating p27 and downregulating CDK2 and cyclin E through its regulatory effects on NRP-1. This is supported by other studies, where overexpression of miR-141 resulted in the suppression of G1-phase cell cycle [19] and inhibited the growth and colony formation of PANC-1 cells [18].

Metastasis is the leading cause of mortality in patients with pancreatic cancer, since clinically non-symptomatic metastasis supervenes from an early stage, greatly impacting the eligibility for surgical resection [41, 42]. Here we have shown that the expression level of NRP-1 is positively correlated with lymph metastasis of pancreatic cancer, underpinning the critical role of NRP-1 in metastasis. In addition, NRP-1 depletion was shown to inhibit the migration of pancreatic cancer cells in vitro, tumor angiogenesis and liver metastasis in animal models. In support, NRP-1 interacts with VEGF to trigger intracellular events leading to the formation of aggressive, invasive and highly vascularized tumors [43]. We have also previously demonstrated that NRP-1 participates in the activation of VEGF/VEGFR2 pathway, which is crucial for tumor angiogenesis by regulating the phosphorylation of focal adhesion kinase (FAK), a key factor in cell migration and metastasis [44, 45].

Numerous studies suggest that EMT contributes to the early-stage dissemination of cancer cells and is pivotal for invasion and metastasis of pancreatic cancer [31]. The activation of the TGF-β pathway promotes EMT through phosphorylating Smad2/3, which together with Smad4 to form a SMAD complex that upregulates the transcription factor Snail [29, 46]. Snail is responsible for the induction of EMT in pancreatic cancer by upregulating N-cadherin and downregulating E-cadherin [28, 47]. On the other hand, NRP-1 performs a co-receptor function for TGF-β and induces the activation of the TGF-β pathway [30]. Here we showed that NRP-1 depletion could inhibit the activation of the TGF-β pathway stimulated by TGF-β ligand, evidenced by the reduced phosphorylation of TGF-βRI, and sequential downregulation of p-Smad2/3, Snail and N-cadherin, and upregulation of E-cadherin. Given the fact that Snail is an important EMT inducer, while E-cadherin downregulation and N-cadherin upregulation are two typical characteristics of EMT [29], the alterations in the expression of these key proteins indicates that NRP-1 depletion could lead to EMT inhibition. Through the process of EMT, epithelial-derived cancer cells acquire the ability to migrate and metastasize [48]; thus such alterations induced by NRP-1 depletion may play a critical role in inhibiting metastasis of pancreatic cancer cells, which was demonstrated in migration assays and liver metastasis animal models in the present study.

In addition, overexpression of miR-141 showed a similar effect as NRP-1 depletion upon the migration and EMT of pancreatic cancer cells through its dysregulation of NRP-1 and regulation on the downstream molecules. In accord, the inhibitory activity of miR-141 on metastasis of pancreatic cancer has been reported previously [19] and downregulation of miR-141 promotes bone metastasis of prostate cancer [35]. In contrast, knockdown of miR-141 inhibited brain metastasis of breast cancer [34]. These inconsistent results indicate that miR-141 may play different regulatory roles on tumor metastasis, depending on the cellular context.

**Fig. 7:**
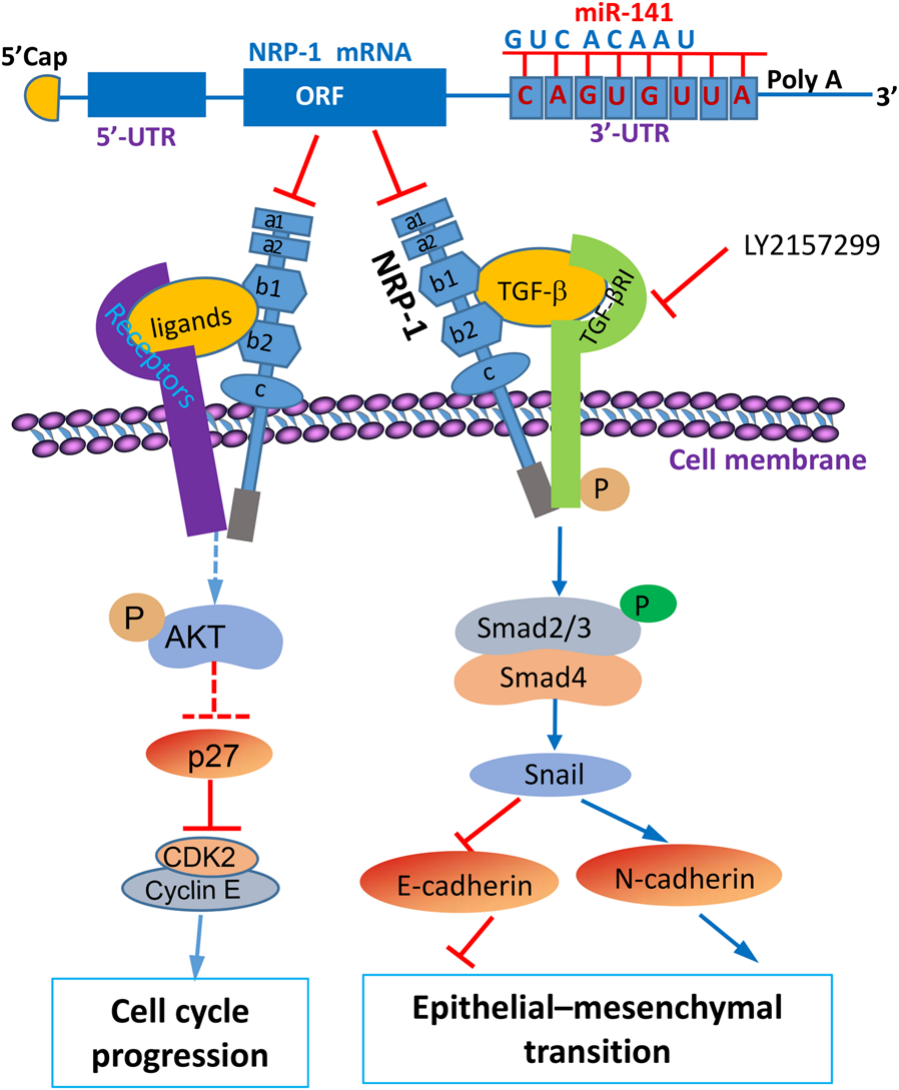
Schematic diagram of the miR-141/NRP-1 axis in regulating EMT and cell cycle progression. MiR-141 negatively regulates NRP-1 by binding to its 3′-UTR. NRP-1 protein consists of 5 extracellular domains (a1, a2, b1, b2 and c), a transmembrane domain and a short cytosolic tail. “→” indicates promotion, positive regulation or activation; “⊥”, inhibition, negative regulation or blockade. A solid line represents identified mechanisms in the present study, while a dotted line, known mechanisms from previously published studies [9, 10]. “p” indicates phosphorylation of proteins. *NRP-1* neuropilin-1, *ORF* open reading frame, *CDK2* cyclin-dependent kinase 2, *TGF-β* transforming growth factor-β, *TGF-βR* TGF-β receptor

The regulatory function and proposed mechanisms of the miR-141/NRP-1 axis on EMT and cell cycle progression of pancreatic cancer cells are summarized schematically in Fig. 7. MiR-141 dysregulates the expression NRP-1 by binding to the 3′UTR region of NRP-1 gene. NRP-1 comprises five extracellular regions of a1a2 domains, b1b2 domains and a c domain as well as a transmembrane domain and a short cytoplasmic tail lacking internal signaling activity [49, 50]. NRP-1 functions as versatile co-receptors that bind to a number of growth factors and couple with cognate receptor tyrosine kinase signaling pathways including VEGF [11, 44, 51], HGF [13] and EGF [12], leading to the enhanced activation of Akt and sequential downregulation of p27 and upregulation of CDK2 and cyclin E, thus enhancing cell cycle progression [9, 10]. NRP-1 co-interacts with TGF-β [30], leading to the phosphorylation of TGF-βRI, which in turn phosphorylates Smad2 and Smad3; phosphorylated Smad2 and Smad3 combine with Smad 4 to form a trimeric SMAD complex that regulates the transcription of the target genes [52]. SMAD complex induces the expression of Snail, which relays TGFβ-activated repression of E-cadherin and upregulation of N-cadherin [46], thus promoting EMT of pancreatic cancer cells. On the other hand, LY2157299, a specific TGF-βR inhibitor [32], blocks the TGF-β pathway, leading to the opposite results of TGF-β ligand.

## Conclusions

The present study has demonstrated that the miR-141/NRP-1 axis is associated with the clinicopathology and contributes to the growth and metastasis of pancreatic cancer. Specifically, NRP-1 is highly expressed in pancreatic cancer tissues and cells that express lower levels of miR-141, whose expression is negatively correlated with NRP-1. By binding to the 3′UTR of NRP-1 gene, miR-141 dysregulates the expression of NRP-1, resulting in inhibition of the proliferative and migratory properties of pancreatic cancer cells and growth of established pancreatic tumors in mice. The expression levels of NRP-1 were positively correlated with tumor grade, lymph metastasis and AJCC staging of pancreatic cancer. NRP-1 depletion inhibited the proliferation, migration and EMT of pancreatic cancer cells, and suppressed their ability to form tumors and to metastasize to livers. NRP-1 depletion and/or miR-141 mimics counteracted the activation of the TGF-β pathway stimulated by TGF-β ligand, resulted in sequential suppression of EMT of pancreatic cancer cells. The present results suggest that the miR-141/NRP-1 axis may be valuable biomarkers and potential therapeutic targets for pancreatic cancer.

## Supplementary information

**Additional file 1.** Supplementary information containing Table S1–2, Materials S1 and Figure S1–8.

## Data Availability

The datasets used and analyzed during the current study are available from the corresponding author on reasonable request.

## Abbreviations

ANOVA: analysis of variance
EdU: 5-ethynyl-2′-deoxyuridine
CDK: cyclin-dependent kinase
EGF: epithelial growth factor
EGFR: epithelial growth factor receptor
ERK: extracellular-signal-regulated kinase
FAK: focal adhesion kinase
FBS: fetal bovine serum
HGF: hepatocyte growth factor
MEK: mitogen-activated protein kinase
MMP-2: matrix metalloproteinase-2
MMP-9: matrix metalloproteinase-9
NRP-1: neuropilin-1
qRT-PCR: quantitative reverse-transcription polymerase chain reaction
VEGF: vascular endothelial growth factor
VEGFR: vascular endothelial growth factor receptor
AJCC: the American Joint Committee on Cancer
DMEM: Dulbecco’s Modified Eagle Medium
UTR: untranslated region
CA19-9: carbohydrate antigen 19-9
EMT: epithelial–mesenchymal transition
TGF-β: transforming growth factor-β
TGF-βR: transforming growth factor-β receptor
miR-141: microRNA-141
