# Supplementary material for: The miR-141/neuropilin-1 axis is associated with the clinicopathology and contributes to the growth and metastasis of pancreatic cancer
Source: Cancer Cell Int. 2019 Sep 27;19:248. doi: 10.1186/s12935-019-0963-2 (PMC6764122; doi:10.1186/s12935-019-0963-2)
Supplement: Supplementary file 1 — Additional file 1. Supplementary information containing Table S1–2, Materials S1 and Figure S1–8. [file 12935_2019_963_MOESM1_ESM.pdf]

Additional File 1 for

The miR-141/Neuropilin-1 axis is associated with clinicopathology and contributes to the growth and metastasis of pancreatic cancer

Lixin Ma,[1] Bo Zhai,[2,3] Huaqiang Zhu,[4] Weidong Li,[2,3] Ziyi Li,[3] Liwang Lei,[1] Shujun Zhang,[2] Haiquan Qiao,[1] Xian Jiang,[1,3,*] Xueying Sun[3,*]

[1] Department of General Surgery, the First Affiliated Hospital of Harbin Medical University, Harbin 150001, China

[2] Department of General Surgery, the Fourth Affiliated Hospital of Harbin Medical University, Harbin 150001, China

[3] The Hepatosplenic Surgery Center, the First Affiliated Hospital of Harbin Medical University, Harbin 150001, China

[4] Department of Hepatobiliary Surgery, Shandong Provincial Hospital, Jinan 250021, China

Contents

Table S1. List of antibodies, main reagents and kits..4

Table S2. Sequence of miRNA mimics, anti-miR-141 and negative control oligonucleotides……………………………………………………………………….....5

Materials S1 5

Immunohistochemistry of clinical specimens………………………………….......5

Establishment of stable transfectants depleted of NRP-1………………………..……6

Transfection of oligonucleotides targeting miRNA-141……………………….......7

Cell viability analysis..7

EdU proliferation assay………………………..7

Assessment of cell cycle………………………...8

Transwell migration assay..8

Cell scratch assay……………………………………………………………..9

In situ hybridization for detecting miR-141……………………………………….9

Quantitative reverse-transcription polymerase chain reaction (qRT-PCR)..............10

Immunoblotting analysis...11

Gelatin zymography assay...11

Immunohistochemistry of assessing gene expression in xenograft tissues………..12

In situ Ki-67 proliferation index……………………………………….........12

Assessment of tumor vascularity...13

References..12

Supplementary Figures...14

Figure S1..14

Figure S2..15

Figure S3..16

Figure S4..17

Figure S5..18

Figure S6..19

Figure S7..20

Figure S8..21

Table S1. List of antibodies, reagents and kits

Name	Catalogue No.	Supplier
Anti-Snail Ab	#3879	CST
Anti-E-Cadherin Ab	#3195	CST
Anti- N-Cadherin Ab	#13116	CST
Anti- Smad2/3 Ab	#8685	CST
Anti-phospho-Smad2 (Ser465/467)/ Smad3 (Ser423/425) Ab	#9510	CST
Anti-Smad4 Ab	#38454	CST
Anti-p27 Ab	#3688	CST
Anti-β-actin Ab	sc-130065	Santa Cruz
Anti- NRP-1 Ab	sc-5307	Santa Cruz
Anti- cyclin E Ab	sc-247	Santa Cruz
Anti-p21 Ab	sc-53870	Santa Cruz
Anti-cyclin D1	sc-8396	Santa Cruz
Anti-MMP-2	sc-13594	Santa Cruz
Anti-MMP-9	sc-21733	Santa Cruz
FITC-conjugated rat anti-mouse Ab	sc-516140	Santa Cruz
Anti-PEA-15 (Ser116) Ab	#PA5-38314	ThermoFisher
Anti-Ki67 Ab	ab15580	Abcam
Anti-TGF-βR I Ab	ab31013	Abcam
Anti- phosphor- TGF-βRI (Ser165) Ab	ab112095	Abcam
Recombinant human TGF-βR 1 protein	ab50036	Abcam
HRP goat anti-mouse Ab	TA130004	OriGene
HRP goat anti-rabbit Ab	TA140003	OriGene
HRP rabbit anti-goat Ab	TA130032	OriGene
HRP rabbit anti-rat Ab	TA130038	OriGene
VECTASTAIN® ELITE ABC kit	SK-4100	Vector
LY2157299	S2230	Selleckchem
TUNEL kit	# 11684795910	Sigma-Aldrich
Lipofectamine2000	#11668019	Invitrogen
CCK-8	CK04-05	Dojindo
DAPI	d9564	Sigma-Aldrich
TRIzol™ Reagent	15596026	ThermoFisher

Note: Ab, primary antibody; CST, Cell Signaling Technology (Boston, MA, USA); Santa Cruz; Santa Cruz Biotechnology (Santa Cruz, CA, USA); Cytoskeleton (Denver, CO, USA); R&D Systems (Minneapolis, MN, USA); Sigma-Aldrich (St. Louis, MO, USA); Invitrogen (Carlsbad, CA, USA), MMP-2, matrix metalloproteinase-2; MMP-9, matrix metalloproteinase-9; FITC, fluorescein isothiocyanate; HRP, horseradish peroxidase; OriGene (OriGene Technologies, Inc., Beijing, China); TUNEL, Terminal deoxynucleotidyl transferase-mediated dUTP nick end labeling agent; Dojindo, Dojindo Molecular Technologies, Gaithersburg, MD, USA; Vector, Vector Laboratories (CA, USA); DAPI, 4',6-diamidino-2-phenylindole.

Table S2. Sequence of miRNA mimics, anti-miR-141 and negative control oligonucleotides

Oligonucleotides		Sequences
**Mimics**	miR-24	5′-UCCGGUGCCUACUGAGCUGAUAUCAGUUCUCAUUUUACACACUGG CUCAGUUCAGCAGGAACAGGAG-3′
	miR-30c	5′-CCAUGCUGUAGUGUGUGUAAACAUCCUACACUCUCAGCUGUGAGC UCAAGGUGGCUGGGAGAGGGUUGUUUACUCCUUCUGCCAUGGA-3′
	miR-124	5′-CUCUCUCCGUGUUCACAGCGGACCUUGAUUUAAAUGUCCAUACAA UUAAGGCACGCGGUGAAUGCCAAGAAUGGGGC-3′
	miR-130a	5′-GGCCAGAGCUCUUUUCACAUUGUGCUACUGUCUGCACCUGUCACU AGCAGUGCAAUGUUAAAAGGGCAUUGGCCG-3′
	miR-141	5′-GGGUCCAUCUUCCAGUACAGUGUUGGAUGGUCUAAUUGUGAAGC UCCUAACACUGUCUGGUAAAGAUGGCUCCCGGG-3′
	miR-148a	5′-GAGGCAAAGUUCUGAGACACUCCGACUCUGAGUAUGAUAGAAGUC AGUGCACUACAGAACUUUGUCUC-3′
	miR-152	5′-GGCCCAGGUUCUGUGAUACACUCCGACUCGGGCUCUGGAGCAGUC AGUGCAUGACAGAACUUGGGCCCGG-3′
	miR-181b	5′-GGUCACAAUCAACAUUCAUUGCUGUCGGUGGGUUGAACUGUGUG GACAAGCUCACUGAACAAUGAAUGCAACUGUGG-3′
	miR-200a	5′-GUGAGCAUCUUACCGGACAGUGCUGGAUUUCCCAGCUUGACUCUA ACACUGUCUGGUAACGAUGUUCAAAGG-3′
	miR-212	5′-GGACAGCGCGCCGGCACCUUGGCUCUAGACUGCUUACUGCCCGGG CCGCCCUCAGUAACAGUCUCCAGUCACGGCCACCG-3′
	miR-320	5′-CCGCCUUCUCUUCCCGGUUCUUCCCGGAGUCGGGAAAAGCUGGGU UGAGAGGGCGAAAAAG-3′
	miR-376b	5′-GGUAUUUAAAACGUGGAUAUUCCUUCUAUGUUUACGUGAUUCCU GGUUAAUCAUAGAGGAAAAUCCAUGUUUUCAG′3
Anti-miR-141		5′-CCAUCUUUACCAGACAGUGUUA-3′
Negative control		5′-CAGUACUUUUGUGUAGUACAA-3′

Materials S1

Immunohistochemistry of clinical specimens

The methods have been described previously [1]. Formalin-fixed and paraffin-embedded specimens were sectioned and mounted on 3-aminopropyltriethoxysilane-coated slides (Sigma, Shanghai, China). The slides were heated in a microwave in 0.01 M citrate buffer for antigen retrieval. Sections were blocked for 2 h and incubated with

a rabbit anti-human NRP-1 Ab (diluted 1:250) at 4ºC overnight. A standard horseradish peroxidase staining procedure was followed using a biotinylated secondary Ab (diluted 1:250) and immunoreactivity developed with Sigma FAST DAB (3,3'-diaminobenzidine tetrahydrochloride) and $CoCl_2$ enhancer tablets. Sections were counterstained with hematoxylin. Normal rabbit sera were diluted 1:10 in PBS, and used for blocking and dilution of Abs. Negative controls were achieved by using irrelevant goat IgG at a dilution of 1:50. NRP-1 staining was assessed in 20 randomly selected fields per specimen using a semi-quantitative grading system, which reflected the proportion and intensity of staining present within the specimen. The staining intensity (Value A) was graded in a four-tier grading system: no staining (0), faint yellow (1), yellow (2) and brown (3). The extent of positive staining (Value B) was determined using a four-tier grading system based on the percentage of positive cells: ≤ 10% (1), 11-40% (2), 41-70% (3), and ≥ 70% (5). The immunohistological score for each specimen was calculated by A × B, and then each specimen was graded low level (≤ 5) and high level (>5).

Establishment of stable transfectants depleted of NRP-1

The NRP-1 shRNA pSuppressorNeo vector targeting NRP-1 gene sequence (GAGAGGUCCUGAAUGUUCC) (corresponding to nucleotides 949-967 of human NRP-1 [GenBank NM_003873.5]) and a scrambled shRNA vector (Sc-shRNA) have been previously described [1, 2]. BxPC-3 cells were seeded in 10-cm plastic dishes and grown to 67% confluence at which point they were transfected with 4 μg of each vector

using Lipofectamine2000 (Invitrogen). They were detached with trypsin after transfection for 48 h, and seeded in selection medium containing geneticin (G418) (500 μg/ml). Stable transfectants were selected at 4 weeks of culture.

Transfection of oligonucleotides targeting miRNA-141

The double-stranded mimics targeting various miRNAs, anti-miR-141 and the negative control (NC) oligonucleotides were purchased from GenePharma Co., Ltd., Shanghai, China). Cells were grown to 60-70% confluence, and incubated with RNAs at a final concentration of 0.1 μM by using Lipofectamine^{TM} 2000 (Invitrogen) in serum-free media for 48 h and then subjected to assays.

Cell viability assay

The Cell Counting Kit-8 (CCK-8) (Dojindo Molecular Technologies, Inc. Beijing, China) was used to determine cell viability. Cells were seeded at 1×10^3 cells /well in 96-well plates. At different time points, the culture medium was replaced with 100 μl of fresh medium containing 10 μl of CCK-8 solution. The cells were further incubated for 2 h at 37°C, and the optical density (OD) at 450 nm was measured. The viability of cells was calculated by using a formula as below: (Experimental OD-Control OD)/Control OD × 100%. The experiment was repeated thrice.

EdU proliferation assay

The methods have been described previously [3]. An EdU (5-ethynyl-2′-deoxyuridine)

proliferation assay was used to assess cell proliferation according to the manufacturer's manual (RiboBio, Guangzhou, China). Cells were plated in 24-well plates at a density of 5×10^4 cells/well and cultured for 48 h. Cells were washed with PBS, and incubated in serum-free DMEM containing 10 μmol/L of EdU for 2 h. EdU-labeled indices were measured by visually scoring nuclei stained with 4',6-diamidino-2-phenylindole (DAPI) in 50 to 100 cells in 20 independent visual fields. EdU-positive cells were scored as a percentage of the total cell number. The experiment was repeated thrice.

Assessment of cell cycle

Cells were seeded at 5.0×10^5 cells/well in 6-well plates, cultured for 48 h, and then harvested. The percentage of cells at G1 and S phases was determined with a cell cycle detection kit (BD Biosciences, Beijing, China) using flow cytometry with a Beckman Coulter Epics Altra II cytometer (Beckman Coulter, California, USA). The experiments were repeated thrice.

Transwell migration assay

Transwell assays were performed using 8-μm pore transwell chambers in 24-well plates BD Bioscience (San Jose, CA, USA). Cells (2×10^4) suspended in 200 μl of serum-free DMEM/F12 medium were seeded on the polycarbonate membrane in a transwell culture chamber and the lower chamber was filled with 800 μl of medium with 10 % FBS. After incubation for 24 h at 37°C in a humidified atmosphere of 5% CO_2, the transwell culture chamber was washed with PBS and the cells on the top surface of the

polycarbonate membrane were removed. Cells that migrated to the bottom surface of the insert were fixed with methanol and stained by Giemsa stain. The cells were counted based on digital images of 5 fields taken randomly at × 200. The experiments were repeated thrice.

Cell scratch assay

Cells were grown in monolayer in 6-well plates to ~95% confluency and treated with mitomycin C to arrest cell growth. Two straight scratches were made with a pipette tip across each cell monolayer. Floating cells were removed by washing with PBS and fresh media. Eight areas of scratches lines were marked for each cell monolayer and imaged by camera, and the scratch distances were again recorded at indicated time points. The original and resulting areas absent of cells were quantified by ImageJ software. The 8 areas of each well were averaged and variation further assessed with duplicate wells for each treatment.

***In situ* hybridization for detecting miR-141**

The *in situ* expression of miR-141 was detected by using previously described methods with appropriate modifications [4, 5]. Double digoxigenin (DIG)-labelled locked nucleic acid probes for miR-141 (ATTGTGACAGACCATTTCTACC, RNA-Tm 84°C), , and a scrambled sequence (GTGTAACACGTCTATACGCCCA, RNA-Tm 87°C) as a negative control (Exiqon, Vedbaek, Denmark) were used according to the manufacturer's manual. Briefly, cells were fixed with 4% paraformaldehyde and

incubated with Proteinase-K (15 μg/mL) for 10 min at 37°C. After being washed twice in phosphate buffered saline (PBS), cells were dehydrated in ethanol, blocked in prehybridization buffer (3% bovine serum albumin [BSA]) for 30 min at 55°C and incubated in hybridization buffer with probes (diluted at 1:2,000) for 1 h at 55°C. They were then washed with standard saline citrate buffer and blocked with 4% BSA for 1 h at room temperature. Positive signals were developed by an overnight incubation with an anti-DIG primary mouse monoclonal antibody at 4°C, followed by incubations with fluorescein isothiocyanate (FITC)-conjugated or Cy3-conjugated secondary antibodies. DAPI (4′,6-diamidino-2-phenylindole) was used to stain cell nuclei. Stained cells were visualized by laser scanning confocal microscopy. All buffers in the above-described experiments were freshly prepared on the day of the experiment.

Quantitative Reverse-Transcription Polymerase Chain Reaction (qRT-PCR)

RNA isolation and quantitative real-time RT-PCR Total RNA, containing miRNA, was extracted from either tissue samples or transfected cells using TRIzol reagent according to the manufacturer's instructions. The reverse transcription was conducted by using TaqMan MicroRNA Reverse Transcription Kit reagents and Reverse Transcription Primers (RT primers) and cDNA was synthesized. The reaction mixtures for qRT-PCR were prepared with the primers for NRP-1 (Forward: 5'-GGAGCTACTGGGCTGTGAAG-3'; and Reverse: 5'-ACCGTATGTCGGGAACTCT G-3') and β-actin (Forward: 5'- AGCGAGCATCCCCCAAAGTT -3'; Reverse: 5'-GGGCACGAAGGCTCATCATT-3'), and analyzed by MX3000P Real-time PCR

systems (Stratagen, USA). The expression of mature miR-141 was performed as above by using a primer (forward, 5'-CAUCUUCCAGUACAGUGUUGGA-3'), and human U6 RNA (with a primer of forward, 5'-CTCGCTTCGGCAGCACA-3') was amplified as an internal control. The reverse primers for U6 and miR-141 were the universal primer provided by Takara. Experiments were performed as triplicate, and the data were calculated by ΔΔCt methods.

Immunoblotting

Protein concentrations of cell or tissue lysates were determined using the Bio-Rad protein assay (Bio-Rad, Richmond, CA, USA). Lysates were resolved on SDS-polyacrylamide gels, and the proteins transferred to PVDF membranes, and immunoblotted as previously described [1, 3, 6-8]. The density of each band was measured using the FR200 densitometric analysis program (Shanghai, China). In preliminary experiments, serial dilutions of lysates containing 2.5, 5, 10, 20, 40 or 80 μg of protein were immunoblotted. Band intensities were measured and plotted against protein amounts to generate a standard curve, and the amount of protein for each immunoblot was determined.

Gelatin zymography assay

The conditioned medium from an equal number of cells that had been incubated in serum-free medium for 48 h was collected and separated on 10% acrylamide gels containing 0.1% gelatin (Invitrogen). Gels were incubated in 2.5% Triton X-100

solution at room temperature with gentle agitation to remove SDS, and soaked in reaction buffer (50 mM Tris–HCl, pH 7.5, 150 mM NaCl, 10 mM $CaCl_2$, and 0.5 mM $ZnCl_2$) at 37°C overnight. After reaction, gels were stained for 1 h with staining solution (0.1% Coomassie Brilliant Blue, 30% methanol, and 10% acetic acid) and then destained in the same solution without Coomassie Brilliant Blue. The gelatinolytic activity of MMP-2 and MMP-9 was visualized as a clear band against a dark background of stained gelatin.

Immunohistochemistry of assessing gene expression in xenograft tissues

Frozen tissues were sectioned (5μm), blocked for 2 h, and incubated with anti-human NRP-1 (diluted 1:250), anti-Ki-67 (diluted 1:800) or anti-CD31 (diluted 1:500) Abs at 4°C overnight. They were subsequently incubated for 30 min with appropriate secondary Abs using the Ultra-Sensitive TMS-P kit (Zhongshan Co., Beijing, China), and immunoreactivity developed with Sigma FAST DAB (3,3'-diaminobenzidine tetrahydrochloride) and $CoCl_2$ enhancer tablets (Sigma-Aldrich, Shanghai, China). Sections were counterstained with hematoxylin, mounted, and examined by microscopy.

In situ Ki-67 proliferation index

The Ki-67 positive cells in immunostained sections as above were counted in 10 randomly selected × 400 high-power fields under microscopy. The Ki-67 proliferation index was calculated according to the following formula: the number of Ki-67 positive cells/ the total cell count × 100%.

Assessment of tumor vascularity

Briefly, 5 μm tumor sections were immunostained with an anti-CD31 Ab as above and examined under microscopy. Stained vessels were counted in ten blindly chosen random fields at 400 × magnification, and the microvessel density was recorded.

References

1. Li, L., et al., *Neuropilin-1 is associated with clinicopathology of gastric cancer and contributes to cell proliferation and migration as multifunctional co-receptors.* J Exp Clin Cancer Res, 2016. **35**(1): p. 16.
2. Wei, Z., et al., *STAT3 interacts with Skp2/p27/p21 pathway to regulate the motility and invasion of gastric cancer cells.* Cell Signal, 2013. **25**(4): p. 931-8.
3. Zhu, H., et al., *Neuropilin-1 regulated by miR-320 contributes to the growth and metastasis of cholangiocarcinoma cells.* Liver Int, 2018. **38**(1): p. 125-135.
4. de Planell-Saguer, M., M.C. Rodicio, and Z. Mourelatos, *Rapid in situ codetection of noncoding RNAs and proteins in cells and formalin-fixed paraffin-embedded tissue sections without protease treatment.* Nat Protoc, 2010. **5**(6): p. 1061-73.
5. Li, W., et al., *LncRNA SNHG1 contributes to sorafenib resistance by activating the Akt pathway and is positively regulated by miR-21 in hepatocellular carcinoma cells.* J Exp Clin Cancer Res, 2019. **38**(1): p. 183.
6. Jiang, W., et al., *Sodium orthovanadate overcomes sorafenib resistance of hepatocellular carcinoma cells by inhibiting Na(+)/K(+)-ATPase activity and hypoxia-inducible pathways.* Sci Rep, 2018. **8**(1): p. 9706.
7. Han, P., et al., *Dual inhibition of Akt and c-Met as a second-line therapy following acquired resistance to sorafenib in hepatocellular carcinoma cells.* Mol Oncol, 2017. **11**(3): p. 320-334.
8. Zhai, B., et al., *Arsenic trioxide potentiates the anti-cancer activities of sorafenib against hepatocellular carcinoma by inhibiting Akt activation.* Tumour Biol, 2015. **36**(4): p. 2323-34.

Supplementary Figures

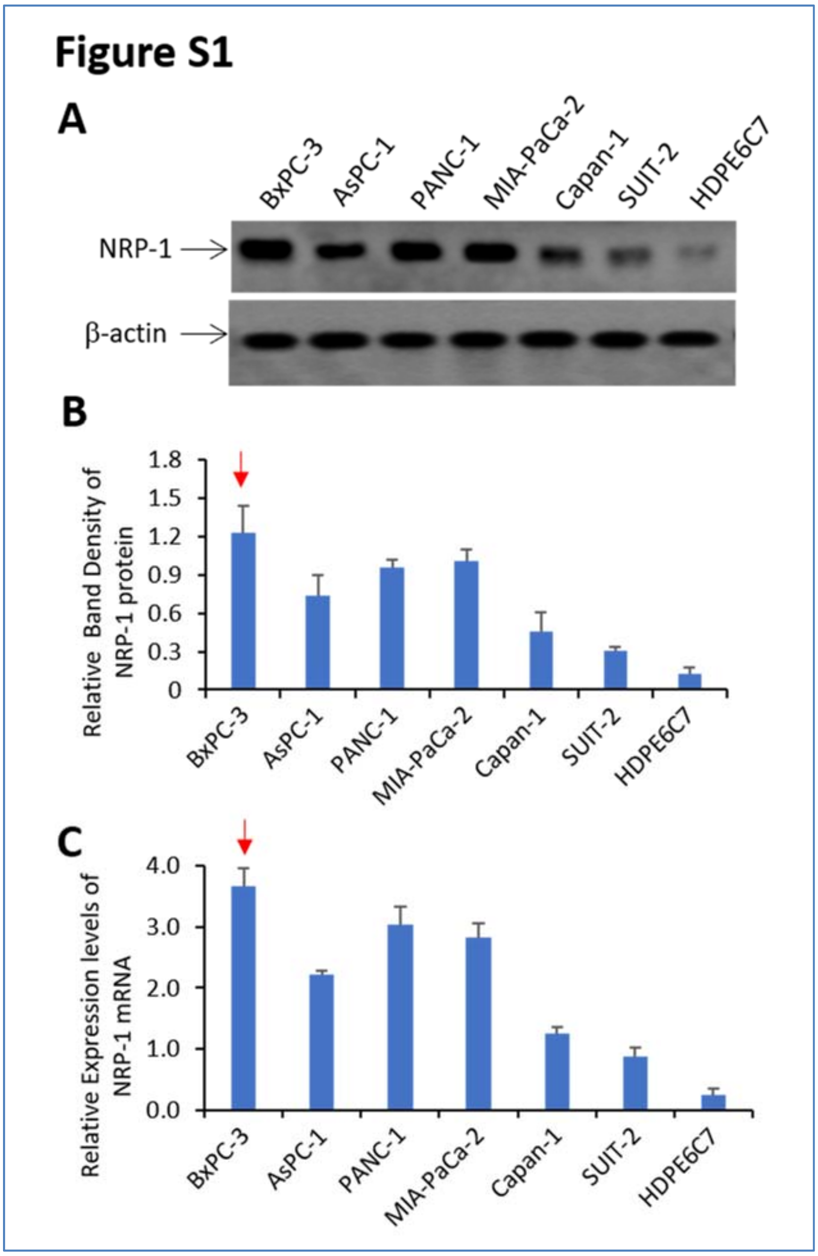

Figure S1. Expression of NRP-1 in human pancreatic cancer cells. (A, B) A panel of human pancreatic cancer cells were lysed and subjected to immunoblotting analysis (A). The density of each band was normalized to β-actin (B). (C) Cells were subjected to qRT-PCR for measuring the expression levels of NRP-1 mRNA. Arrow points to cells expressed the highest levels of NRP-1.

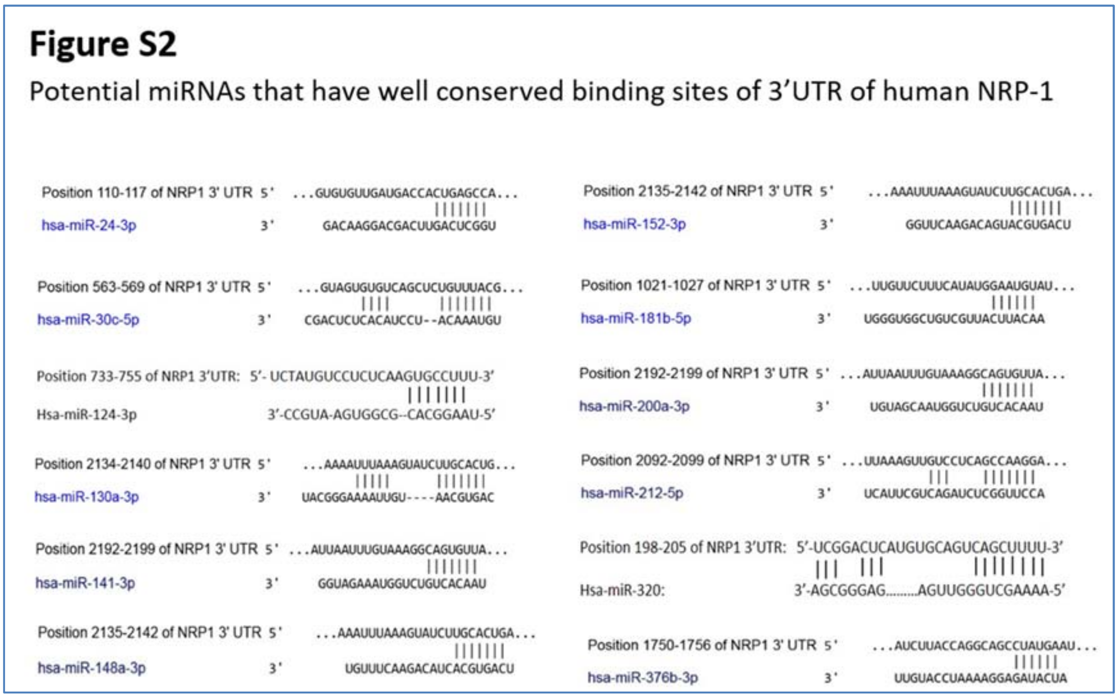

Figure S2. Predicted binding sites of Hsa-miRs to the 3’-UTR of the human NRP-1 gene. Twelve potential miRNAs contain respective binding sites to the 3’-UTR of the human NRP-1 gene as analyzed by using miRNA target prediction programs, including miRWalk (http://mirwalk.umm.uni-heidelberg.de/), TargetScan (http://www.targetscan.org/), miRanda (https://omictools.com/miranda-tool), miRTarBase (http://mirtarbase.mbc.nctu.edu.tw/) and mirdb (http://mirdb.org/).

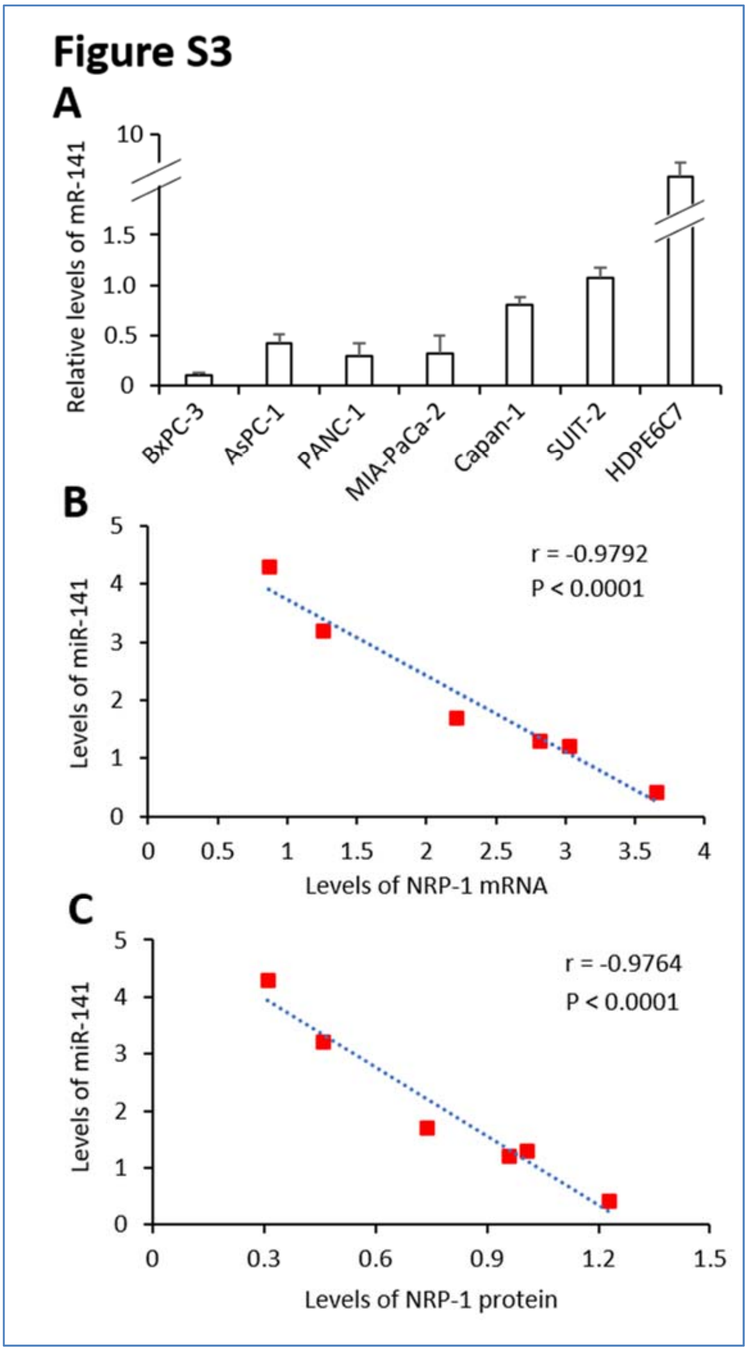

Figure S3. The expression levels of miR-141 is negatively correlated with NRP-1 expression in pancreatic cancer cells. (A) A panel of human pancreatic cancer cells were subjected to qRT-PCR for measuring the expression levels of miR-141, whose correlation with NRP-1 protein (as shown in Fig. S1B) (B) and mRNA (as shown in Fig. SC) (C) were analyzed by using a Pearson test, and the correlation coefficient is denoted by “r”.

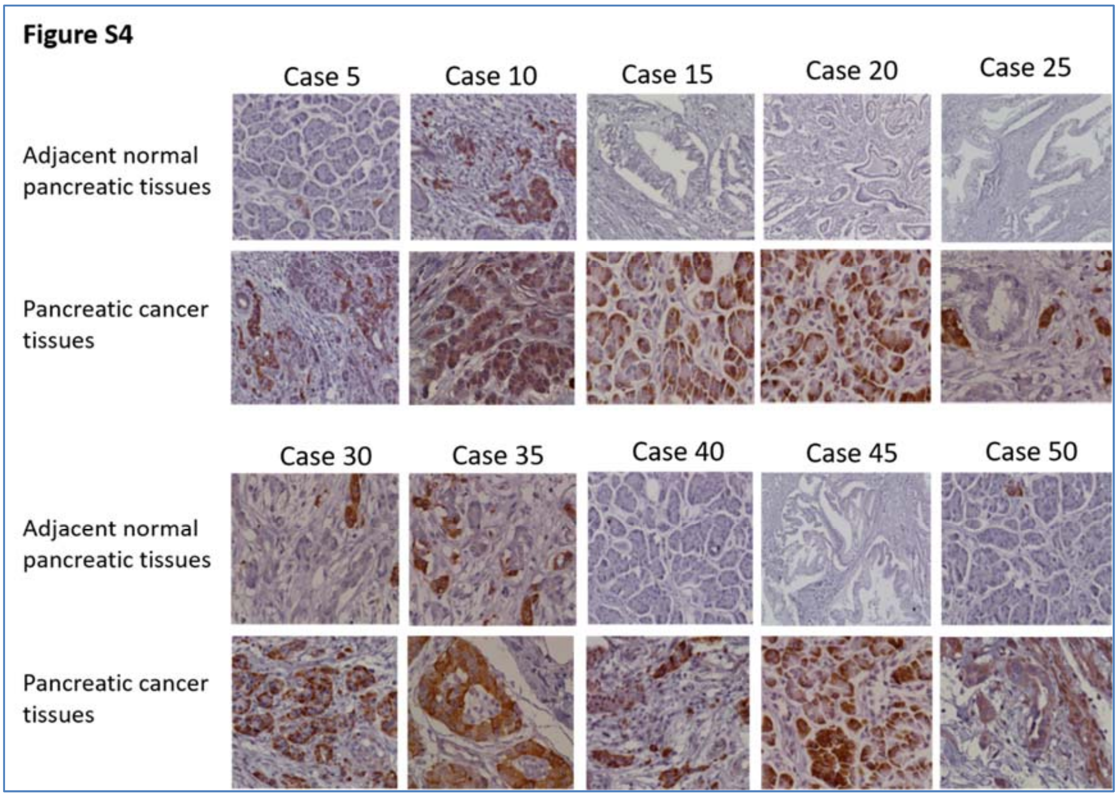

Figure S4. Immunohistochemistry analysis of NRP-1 expression in clinical specimens. Representative images are sections prepared from pancreatic cancer tissues and the corresponding adjacent normal pancreatic tissues, which were immunostained by an anti-NRP-1 Ab (magnification × 200).

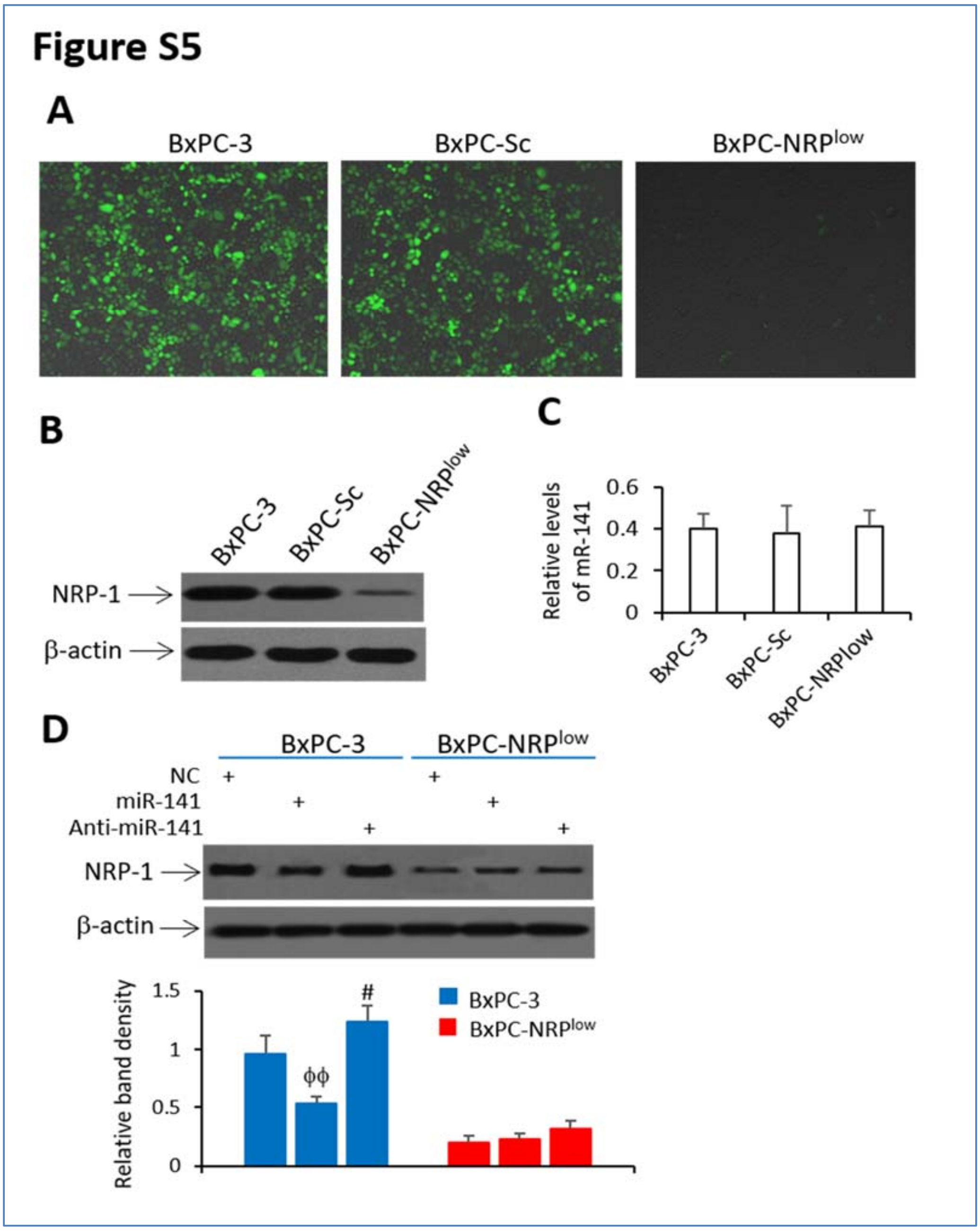

Figure S5. Expression of NRP-1 and miR-141 in BxPC-3, BxPC-Sc and BxPC-NRP^{low} cells. (A, B) The expression of NRP-1 was examined by fluorescent immunohistochemistry (A) and immunoblotting (B) with an anti-NRP-1 Ab. (C) The expression of miR-141 was analyzed by qRT-PCR. (D) BxPC-3 and BxPC-NRP^{low} cells were transfected with negative control (NC), miR-141 mimics or anti-miR-141 oligonucleotides for 48 h and then subjected to immunoblotting. "ϕϕ" (P<0.001) indicates a significant reduction, while "#" (P<0.05), a significant increase, compared with NC-transfected BxPC-3 cells.

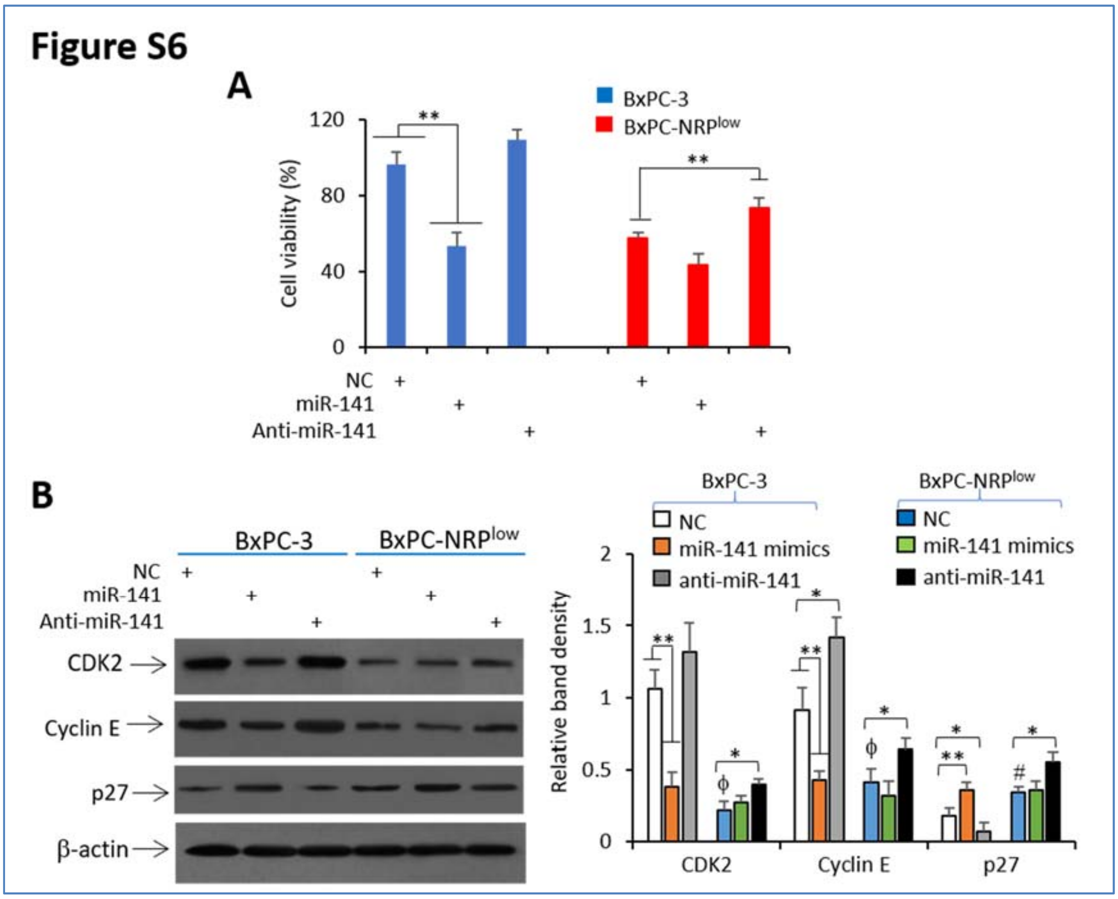

Figure S6. MiR-141 inhibits cell proliferation via downregulating NRP-1. BxPC-3 and BxPC-NRP^{low} cells transfected with negative control (NC), miR-141 mimics or anti-miR-141 oligonucleotides were cultured for 48 hours. (A) Cell viability was measured and normalized to untreated BxPC-3 cells. (B) The above cells were subjected to immunoblotting. "*" ($P<0.05$) and "**" ($P<0.001$) indicate a significant difference. "ϕ" ($P<0.05$) indicates a significant reduction, while "#" ($P<0.05$), a significant increase, from NC-treated BxPC-3 cells.

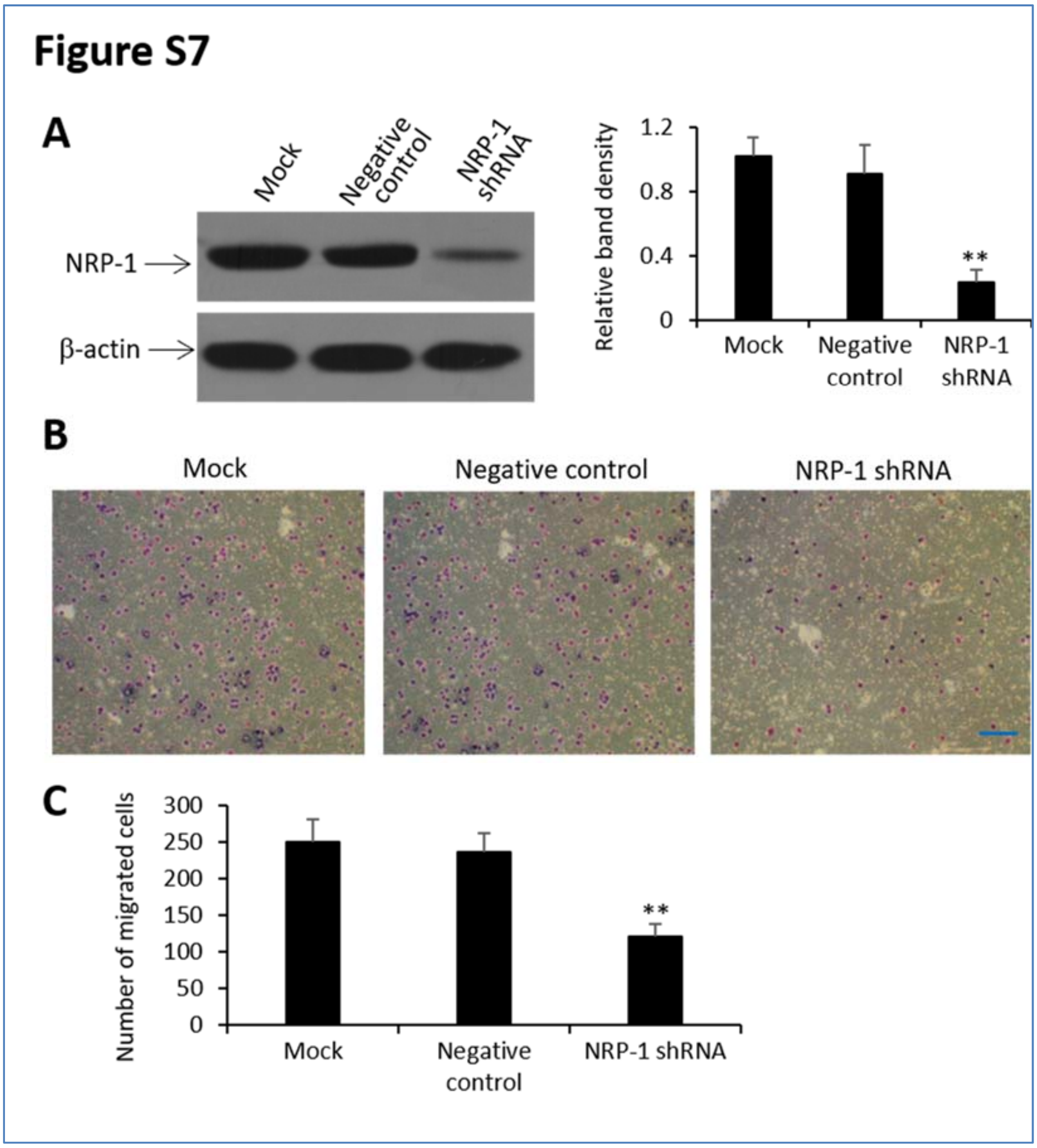

Figure S7. Depletion of NRP-1 inhibits the migrating ability of PANC-1 cells. PANC-1 cells were either mock-transfected or transfected with negative control or NRP-1 shRNA for 48 hours. (A) The above cells were subjected to immunoblotting and the density of each band was normalized to β-actin. (B, C) The above cells were subjected to Transwell migration assays. (A) Migrated cells were visualized using Giemsa staining. Magnification bar = 100 μm. (B) Numbers of migrating cells were counted. "**" (P<0.001) indicates a significant difference from mock-treated cells.

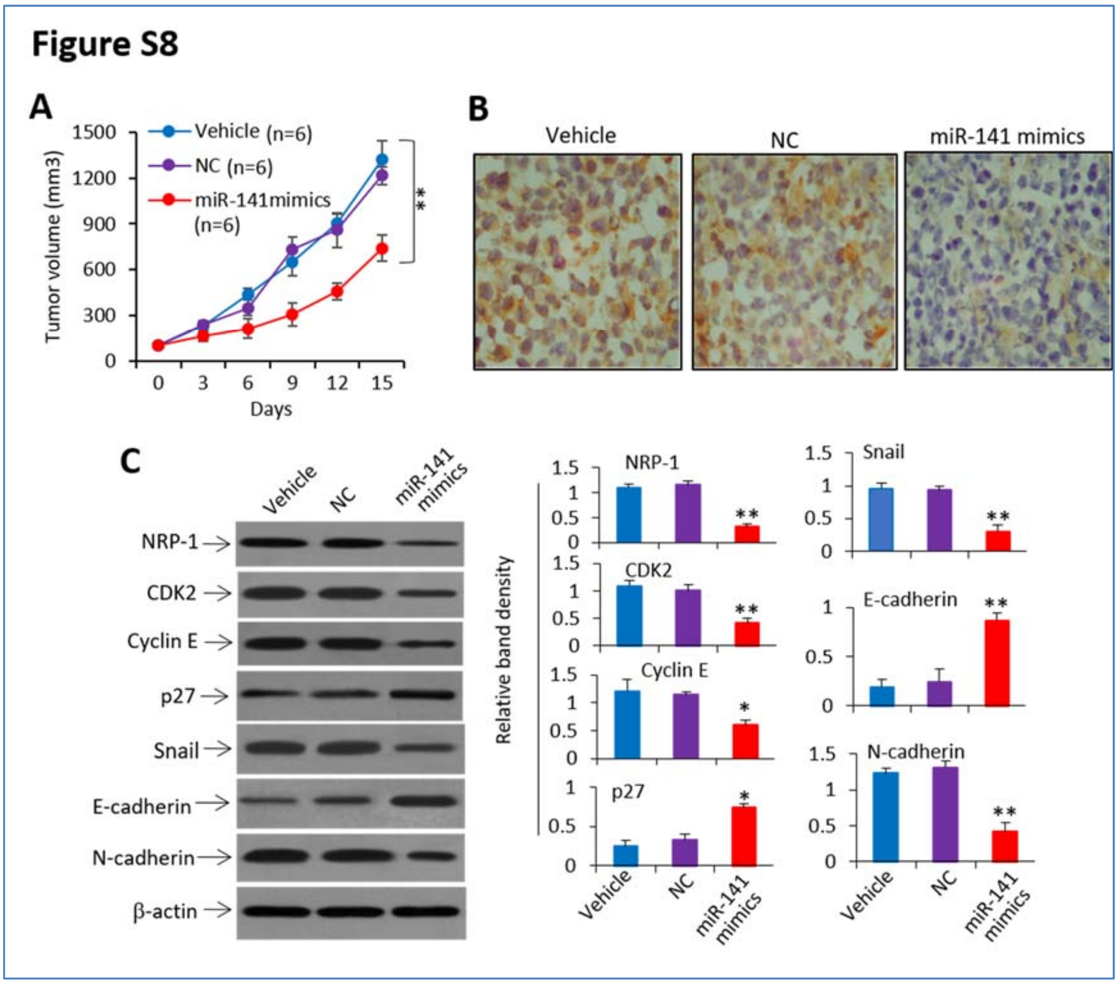

Figure S8. MiR-141 inhibits the growth of pancreatic tumors by regulating NRP-1 *in vivo*. (A) PANC-1 tumors were established in mice, and injected with vehicle, negative control (NC) oligonucleotides or miR-141 mimics when they reached ~100 mm^3. The sizes of tumors were monitored. (B) Illustrated are representative tumor sections prepared 4 days following intratumoral injection, and immunostained brown by an anti-NRP-1 Ab. Magnification × 400. (C) Tumor homogenates were immunoblotted for detecting key proteins regulated by NRP-1-related pathways. "n", number of mice. "*" indicates P<0.05, and "**", P<0.001, from vehicle-treated tumors.
